# Supplementary material for: Legionella pneumophila modulates host energy metabolism by ADP-ribosylation of ADP/ATP translocases
Source: eLife. 2022 Jan 27;11:e73611. doi: 10.7554/eLife.73611 (PMC8820735; doi:10.7554/eLife.73611)
Supplement: Supplementary file 1. — The protein band specifically present in Af1521 pulldown samples from expressing Ceg3 was analyzed by mass spectrometry; the 10 proteins with the most hits were listed. [file elife-73611-supp1.docx]

**Identification of ADP/ATP translocases as the targets of Ceg3 in samples obtained by Af1521-pulldown.** The protein band specifically present in Af1521 pulldown samples from expressing Ceg3 was analyzed by mass spectrometry; the 10 proteins with the most hits were listed.

| **Rank** | **Gene Name** | **Protein Description** | **Spectral Counts** |
| --- | --- | --- | --- |
| 1 | KRT1 | Keratin 1 | 232 |
| 2 | SLC25A6 | ADP/ATP translocase 3 | 87 |
| 3 | SLC25A5 | ADP/ATP translocase 2 | 84 |
| 4 | SLC25A4 | ADP/ATP translocase 1 | 71 |
| 5 | KRT10 | Keratin, type I cytoskeletal 10 | 64 |
| 6 | KRT2 | Keratin, type II cytoskeletal 2 | 57 |
| 7 | KRT9 | Keratin, type I cytoskeletal 9 | 56 |
| 8 | SLC25A31 | ADP/ATP translocase 4 | 40 |
| 9 | KRT9 | Keratin, type I cytoskeletal 9 | 36 |
| 10 | KRT13 | Keratin, type I cytoskeletal 13 | 29 |
