# Supplementary material for: Legionella pneumophila modulates host energy metabolism by ADP-ribosylation of ADP/ATP translocases
Source: eLife. 2022 Jan 27;11:e73611. doi: 10.7554/eLife.73611 (PMC8820735; doi:10.7554/eLife.73611)
Supplement: Supplementary file 2. [file elife-73611-supp2.docx]

**Bacterial strains, antibodies, plasmids and primers used in this study**

| Bacterial Strains | Source | Identifier |
| --- | --- | --- |
| *L. pneumophila* (Philadelphia-1) LP02 | (Berger and Isberg, 1993) | N/A |
| *L. pneumophila* LP03 | (Berger and Isberg, 1993) | N/A |
| LP02Δ*ceg3* | This study | N/A |
| LP02Δ*ceg3* (pZL507) | This study | N/A |
| LP02Δ*ceg3* (pZLQ-Flag) | This study | N/A |
| LP02Δ*ceg3* (pCeg3) | This study | N/A |
| LP02Δ*ceg3* (pCeg3_E141A/E143A_) | This study | N/A |
| LP02 (pCeg3) | This study | N/A |
| LP03 (pCeg3) | This study | N/A |
| *E.coli* BL21(DE3) | NEB | CAT#C2527I |
| *E.coli* XL1-Blue | Agilent | CAT#200249 |

| Antibodies | Source | Identifier |
| --- | --- | --- |
| anti-HA | Sigma | cat# H3663 |
| anti-Flag | Sigma | cat# F1804 |
| anti-ICDH | (Xu et al., 2010) | N/A |
| anti-tubulin | DSHB | E7 |
| anti-PDHA1 | Proteintech | cat# 18068-1-AP |
| anti-ATPB | Proteintech | cat# 17247-1-AP |
| anti-TOM20 | Proteintech | cat# 11802-1-AP |
| anti-VDAC1 | Proteintech | cat# 55259-1-AP |
| anti-Cyto c | Santa Cruz | cat# sc-13560 |
| anti-Calnexin | Proteintech | cat# 10427-2-AP |
| anti-ANT1/2 | Proteintech | cat# 17796-1-AP |
| anti-GM130 | BD Biosciences | cat# 610822 |
| anti-ADPR | Sigma | cat# MABE1016 |

| Plasmids | Source | Identifier |
| --- | --- | --- |
| pZL507 | (Xu et al., 2010) | N/A |
| pZLQ-Falg::*ceg3* | This study | N/A |
| pZLQ-Flag::*ceg3_E141A/E143A_* | This study | N/A |
| p4xFlagCMV | (Qiu et al., 2016) | N/A |
| p4xFlagCMV::*ceg3* | This study | N/A |
| p4xFlagCMV::*ceg3_E141A/E143A_* | This study | N/A |
| pAPH-HA | This study | N/A |
| pAPH-HA::*ceg3* | This study | N/A |
| pAPH-HA::*ceg3_E141A/E143A_* | This study | N/A |
| pCDNA3.1::*ceg3-Flag* | This study | N/A |
| GFP | (Qiu et al., 2016) | N/A |
| GFP::*ceg3* | This study | N/A |
| GFP::*ceg3_E141A/E143A_* | This study | N/A |
| pQE30 | Qiagen | CAT#32915 |
| pQE30::*ceg3* | This study | N/A |
| pQE30::*ceg3_E141A/E143A_* | This study | N/A |
| pGST | Cytiva | CAT#28954648 |
| pGST::*ceg3* | This study | N/A |
| pGST::*ceg3_E141A/E143A_* | This study | N/A |
| pETSumo::*ceg3* | This study | N/A |
| pQE30::*ceg3∆C20* | This study | N/A |
| pQE30::*ceg3∆C40* | This study | N/A |
| pQE30::*ceg3∆C60* | This study | N/A |
| pQE30::*ceg3∆C80* | This study | N/A |
| pQE30::*ceg3∆N20* | This study | N/A |
| pQE30::*ceg3∆N20∆C40* | This study | N/A |
| pQE30::*ceg3∆N20∆C80* | This study | N/A |
| p4xFlagCMV::*mavC* | (Gan et al., 2018) | N/A |
| p4xFlagCMV::*ANT1* | This study | N/A |
| p4xFlagCMV::*ANT1_V227K/R237K_* | This study | N/A |
| p4xFlagCMV::*ANT2* | This study | N/A |
| p4xFlagCMV::*ANT3* | This study | N/A |
| p4xFlagCMV::*ANT4* | This study | N/A |
| pYES1NTA | Invitrogen | Cat#V825220 |
| pYES1NTA::*ceg3* | This study | N/A |
| pYES1NTA::*ceg3_E141A/E143A_* | This study | N/A |
| RFP::*ANT1* | This study | N/A |
| p4xFlagCMV::*ANT1_R60K_* | This study | N/A |
| p4xFlagCMV::*ANT1_R72K_* | This study | N/A |
| p4xFlagCMV::*ANT1_R138K_* | This study | N/A |
| p4xFlagCMV::*ANT1_R140K_* | This study | N/A |
| p4xFlagCMV::*ANT1_R152K_* | This study | N/A |
| p4xFlagCMV::*ANT1_R235K_* | This study | N/A |
| p4xFlagCMV::*ANT1_R236K_* | This study | N/A |
| p4xFlagCMV::*ANT1_R237K_* | This study | N/A |
| pCDH-CMV-MCS-EF1a-RFP | System Biosciences | Cat#CD512B-1 |
| pCDH-CMV-MCS-EF1a-RFP:: *ceg3* | This study | N/A |
| pCDH-CMV-MCS-EF1a-RFP::*ceg3_E141A/E143A_* | This study | N/A |
| pMD2.G | Addgene | Cat#12259 |
| psPAX2 | Addgene | Cat#12260 |

| Primers | Sequence (Restriction enzyme sites are underlined) | Note |
| --- | --- | --- |
| pSL1001 | ctaggatccatgaacagattaaaatttttttc | *ceg3* 5F BamHI |
| pSL1002 | catgtcgacttaaactctaggggataatg | *ceg3* 3R SalI |
| pSL1003 | catgtcgacgctggttattgcttggtgat | *ceg3* up SalI  knockout |
| pSL1004 | ctaggatccaagtaacgcattgtgaaatttg | *ceg3* up BamHI knockout |
| pSL1005 | ctaggatccgttacttattatgtaaaggaatc | *ceg3* down BamHI knockout |
| pSL1006 | catgagctcgcttaaagccactgcggttg | *ceg3* down SacI knockout |
| pSL1007 | atcgacaaccagttctgcttttgccccgtcatgctcacc | *ceg3 _E141A/E143A_* -1 |
| pSL1008 | ggtgagcatgacggggcaaaagcagaactggttgtcgat | *ceg3 _E141A/E143A_* -2 |
| pSL1009 | ctagctagcgccaccatgaacagattaaaatttttttc | *ceg3* 5F NheI |
| pSL1010 | catggatccttacttatcgtcgtcatccttgtaatcaactctaggggataatgaa | *ceg3* 3R BamHI |
| pSL1011 | ctaggatccatgatttctaatttaagcgatcc | *ceg3∆N20* 5F BamHI |
| pSL1012 | catgtcgacttaatctccgtcaaatcgcctc | *ceg3∆C20* 3R SalI |
| pSL1013 | catgtcgacttaaagcttggcaaactcttc | *ceg3∆C40* 3R SalI |
| pSL1014 | catgtagacttacgaactgaaatcaccaagtt | *ceg3∆C60* 3R SalI |
| pSL1015 | catgtcgacttaattgggaaccactccccca | *ceg3∆C80* 3R SalI |
| pSL1016 | ctaggatccggtgatcacgcttggagct | *ANT1* 5F BamHI |
| pSL1017 | catgtcgacttagacatattttttgatctcat | *ANT1* 3R SalI |
| pSL1018 | ctatgatcaacagatgccgctgtgtcct | *ANT2* 5F BclI |
| pSL1019 | catgtcgacttatgtgtacttcttgatttca | *ANT2* 3R SalI |
| pSL1020 | ctaggatccacggaacaggccatctcct | *ANT3* 5F BamHI |
| pSL1021 | catctcgagttagatcaccttcttgagctc | *ANT3* 3R XhoI |
| pSL1022 | ctaggatcccatcgtgagcctgcgaaaaag | *ANT4* 5F BamHI |
| pSL1023 | catgtcgacttacctaccaccaatatcaata | *ANT4* 3R SalI |
| pSL1024 | cattgattgtgtggtgaaaatccctaaggagcagg | *ANT1_R60K_* -1 |
| pSL1025 | cctgctccttagggattttcaccacacaatcaatg | *ANT1_R60K_* -2 |
| pSL1026 | cttcctctccttctggaagggtaacctggc | *ANT1_R72K_* -1 |
| pSL1027 | gccaggttacccttccagaaggagaggaag | *ANT1_R72K_* -2 |
| pSL1028 | acccgctggactttgctaagaccaggttgg | *ANT1_R138K_* -1 |
| pSL1029 | ccaacctggtcttagcaaagtccagcgggt | *ANT1_R138K_* -2 |
| pSL1030 | gactttgctaggaccaagttggctgctgatgtg | *ANT1_R140K_* -1 |
| pSL1031 | cacatcagcagccaacttggtcctagcaaagtc | *ANT1_R140K_* -2 |
| pSL1032 | caagggcgccgcccagaaggagttccatggtctgg | *ANT1_R152K_* -1 |
| pSL1033 | ccagaccatggaactccttctgggcggcgcccttg | *ANT1_R152K_* -2 |
| pSL1034 | tcctacccctttgacactgttaagcgtagaatgatgatgcag | *ANT1_R235K_* -1 |
| pSL1035 | ctgcatcatcattctacgcttaacagtgtcaaaggggtagga | *ANT1_R235K_* -2 |
| pSL1036 | tacccctttgacactgttcgtaagagaatgatgatgcagtc | *ANT1_R236K_* -1 |
| pSL1037 | gactgcatcatcattctcttacgaacagtgtcaaaggggta | *ANT1_R236K_* -2 |
| pSL1038 | tgacactgttcgtcgtaaaatgatgatgcagtccg | *ANT1_R237K_* -1 |
| pSL1039 | cggactgcatcatcattttacgacgaacagtgtca | *ANT1_R237K_* -2 |
| pSL1040 | gcagtcgcagggctgaagtcctacccctttga | *ANT1_V227K_* -1 |
| pSL1041 | tcaaaggggtaggacttcagccctgcgactgc | *ANT1_V227K_* -2 |
