# Supplementary material for: Legionella pneumophila modulates host energy metabolism by ADP-ribosylation of ADP/ATP translocases
Source: eLife. 2022 Jan 27;11:e73611. doi: 10.7554/eLife.73611 (PMC8820735; doi:10.7554/eLife.73611)

Figure 1E source data

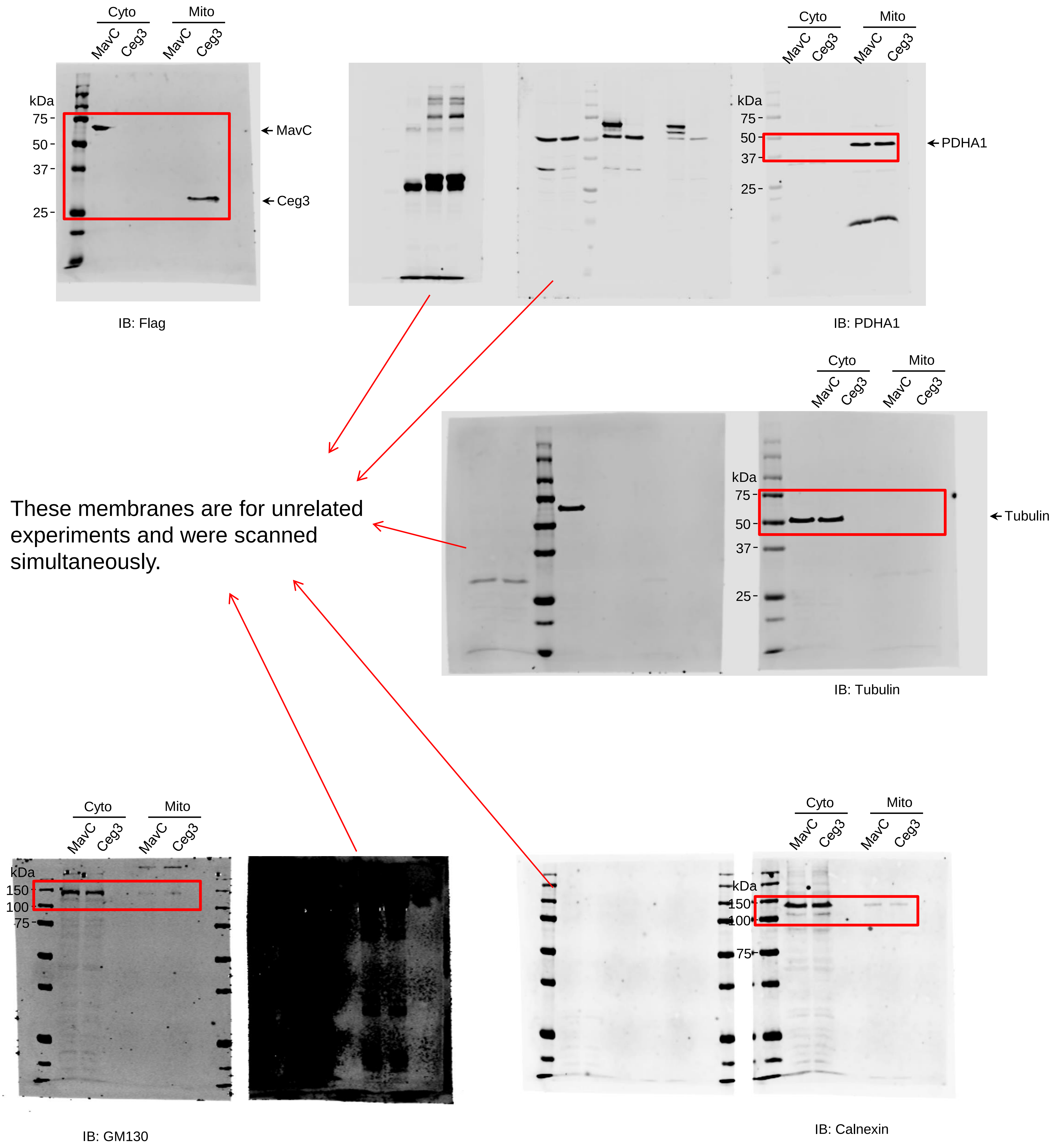

Figure 1F source data

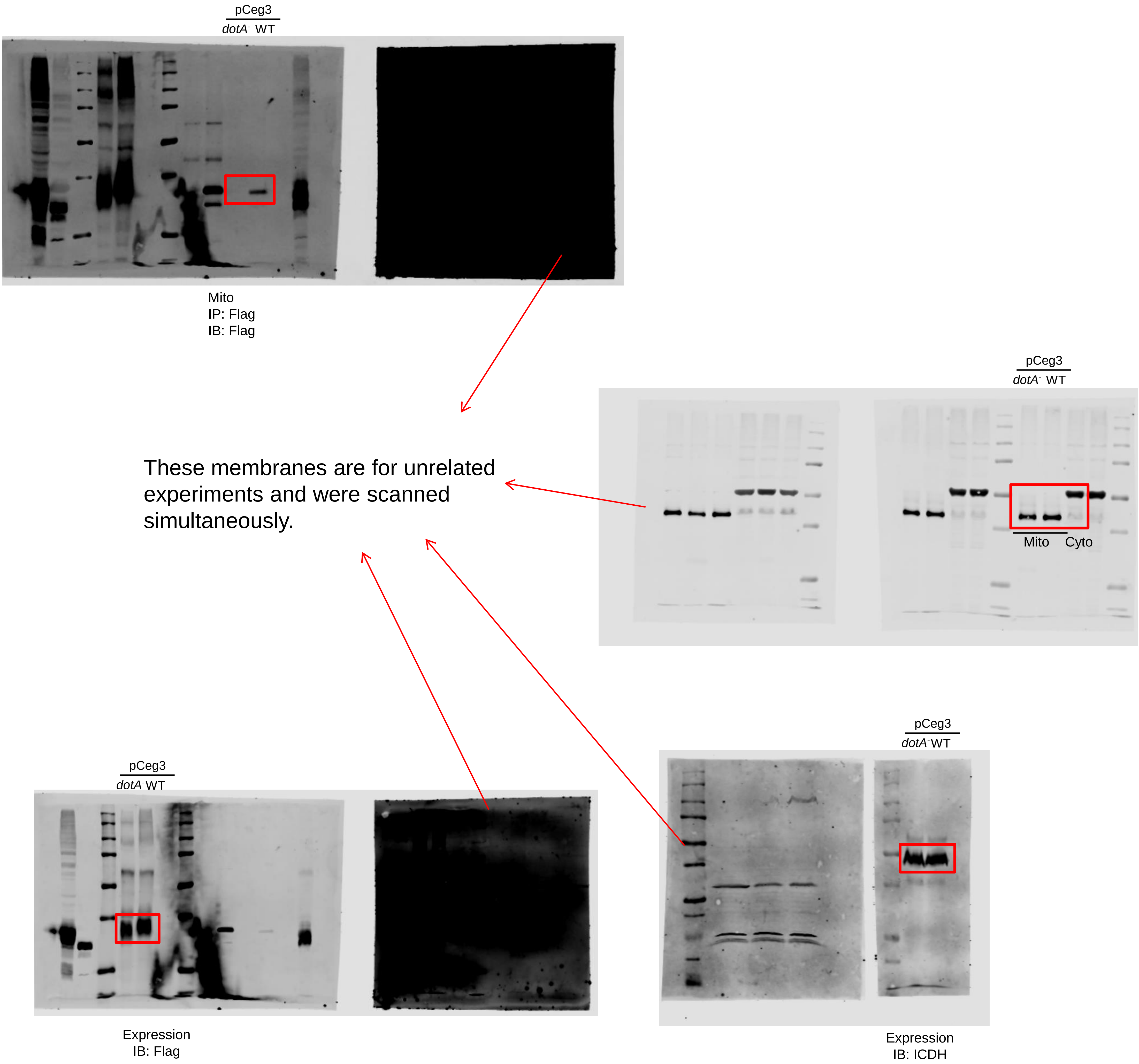

Figure 1G source data

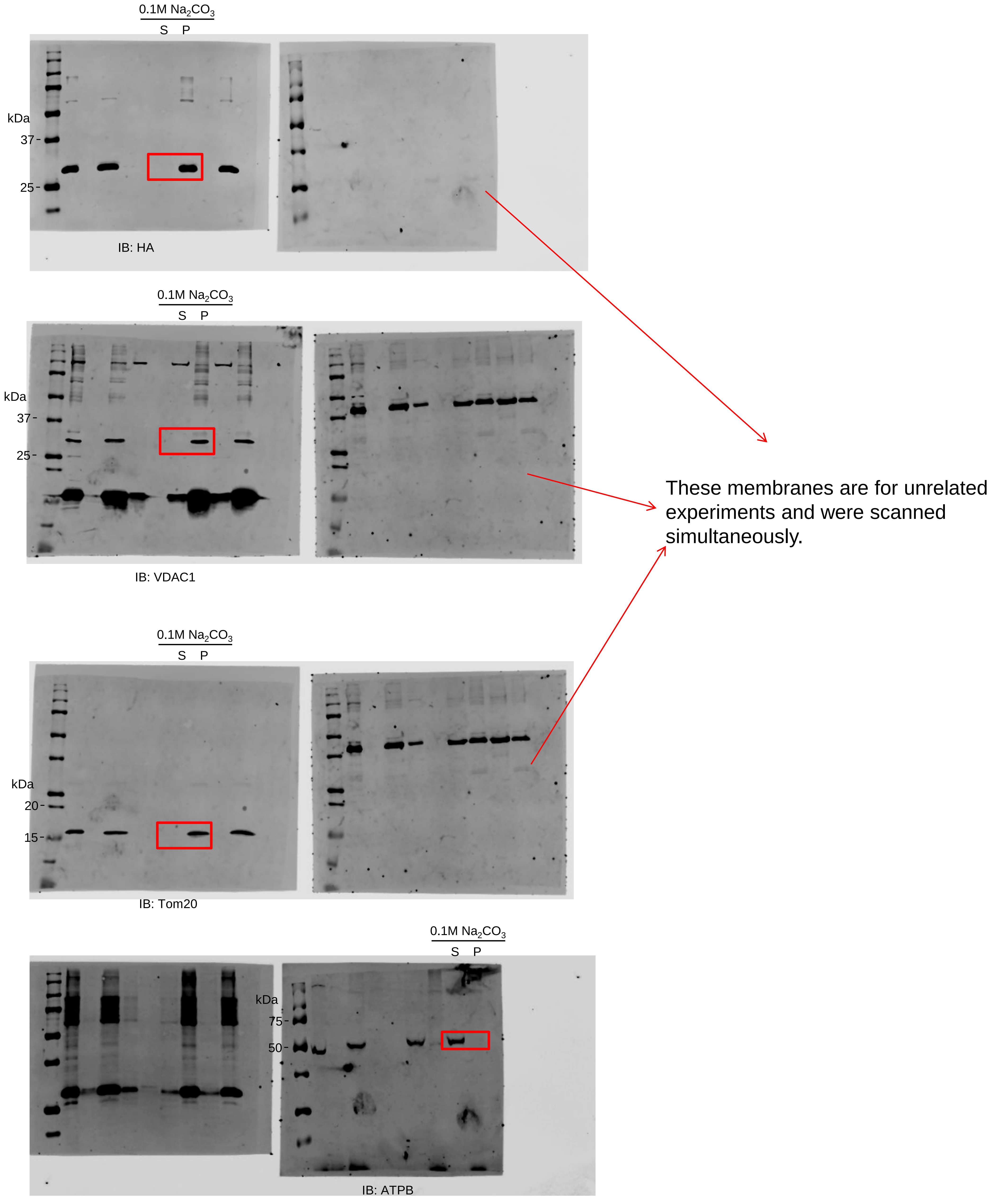

Figure 1G source data

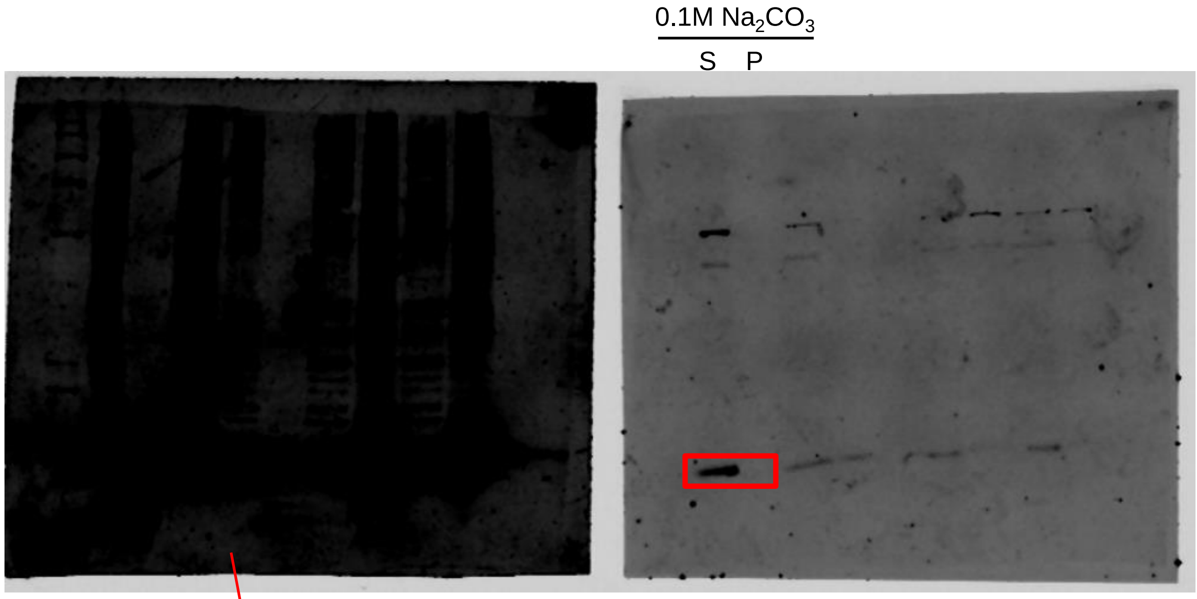

IB: Cyto C

This membrane is for an unrelated experiment and was scanned simultaneously.

Figure 2A source data

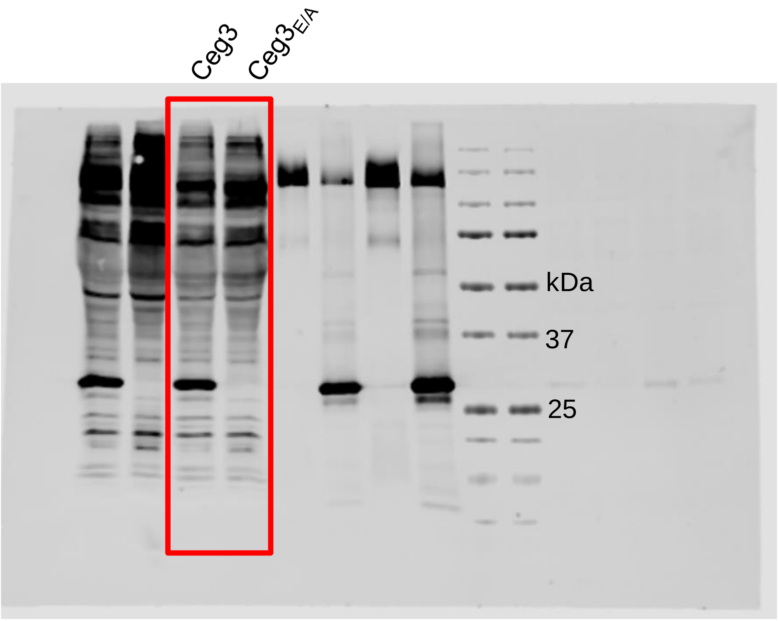

IB: ADPR

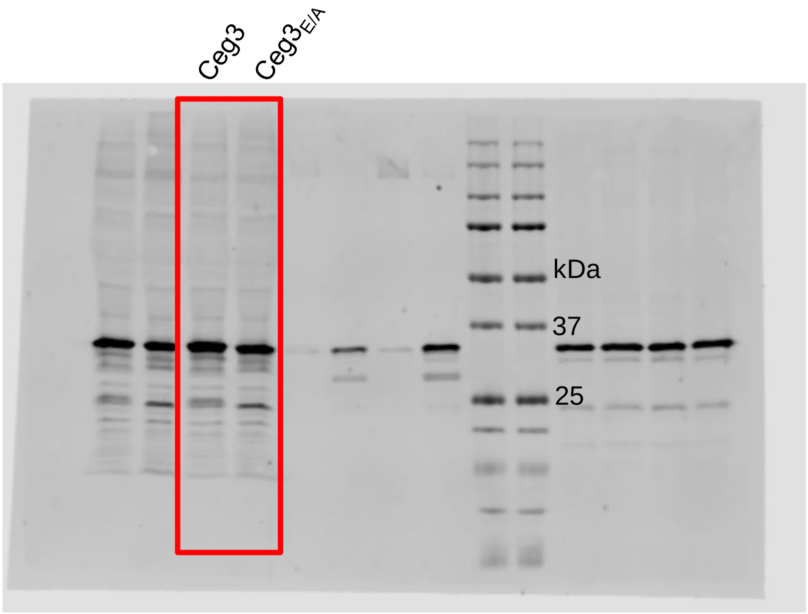

IB: Flag

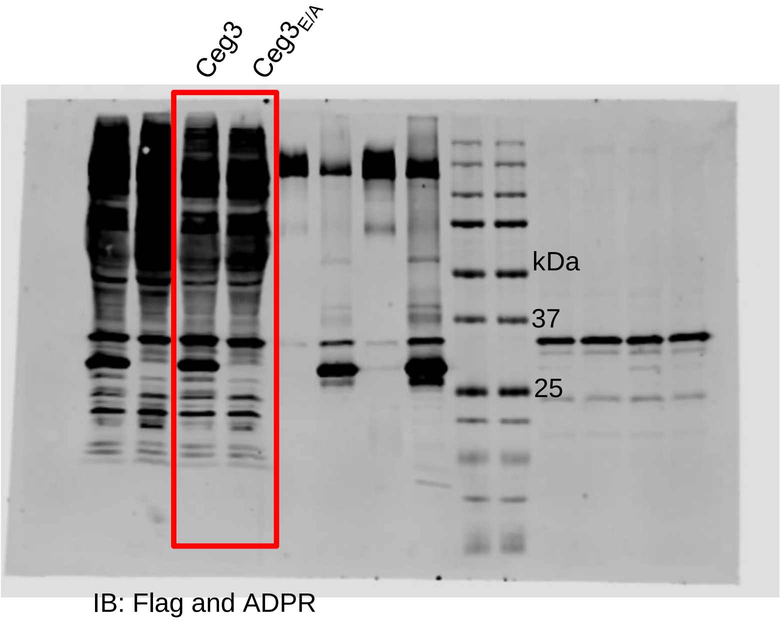

IB: Flag and ADPR

Figure 2B source data

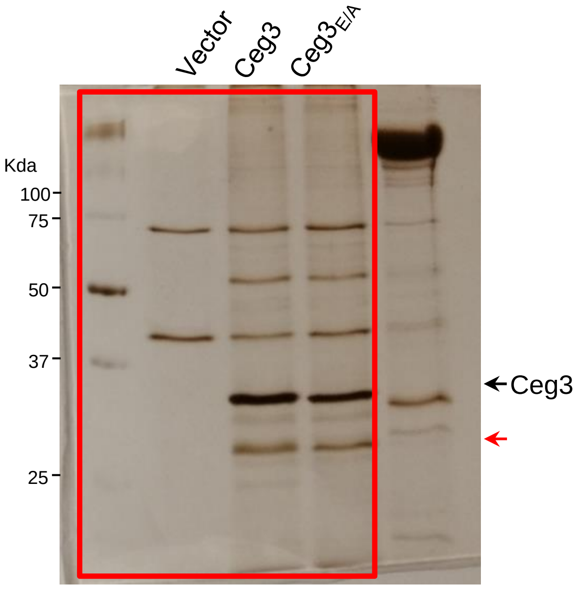

IP:Flag
Silver staining

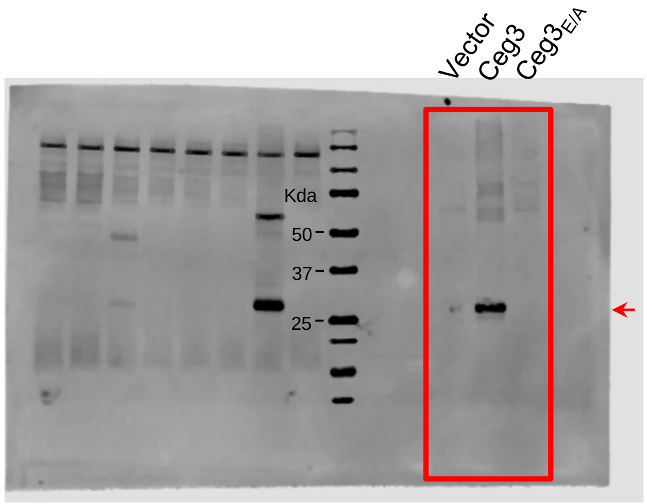

IP:Flag IB: ADPR

Figure 2C source data

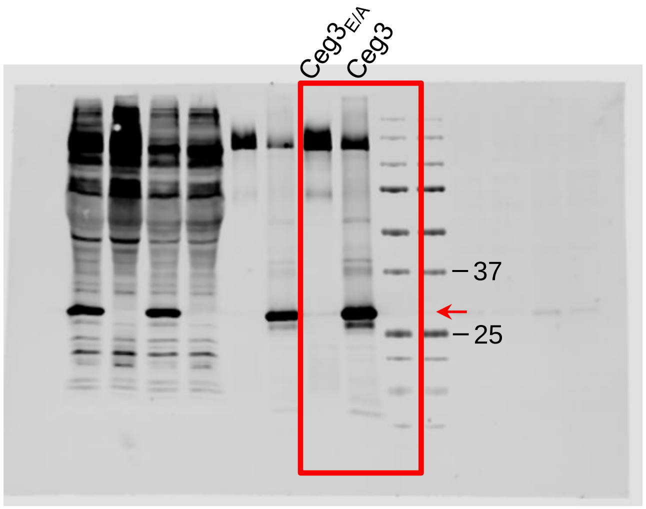

PD: Af1521
IB: ADPR

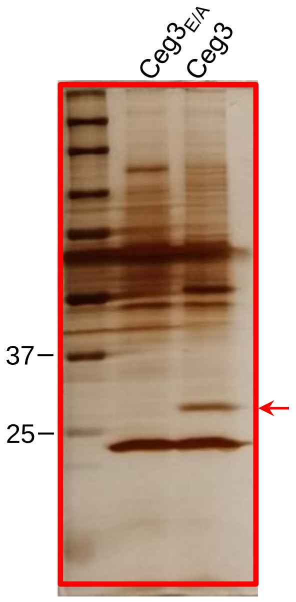

Figure 2D source data

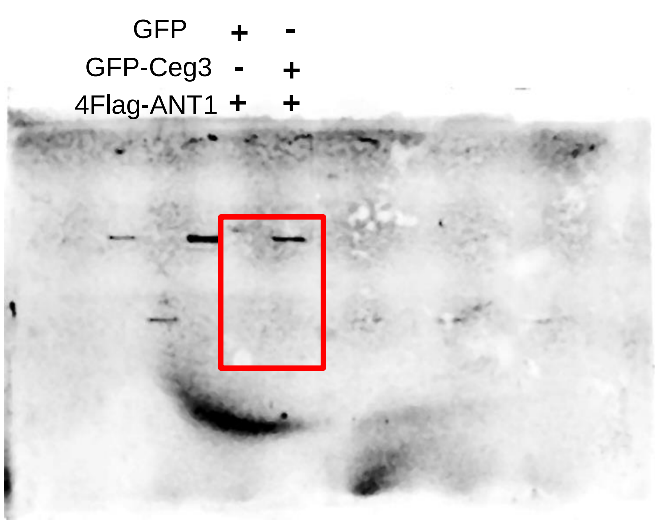

IP:Flag
IB:GFP

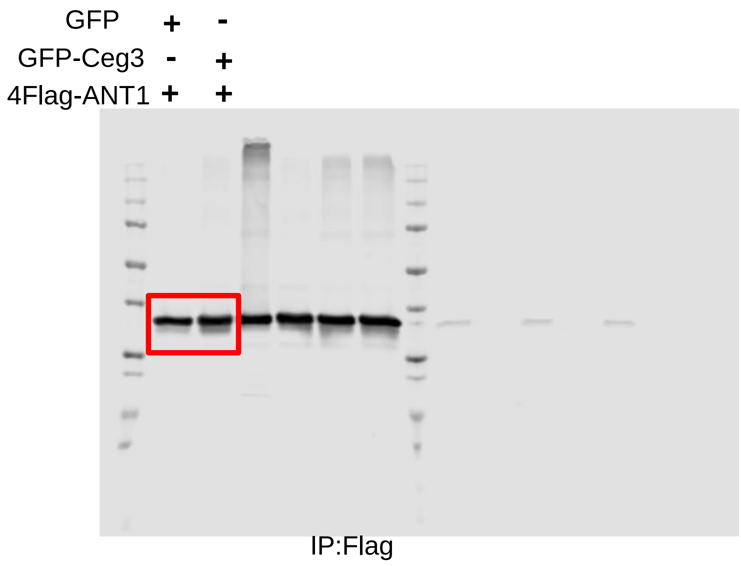

IP:Flag
IB:Flag

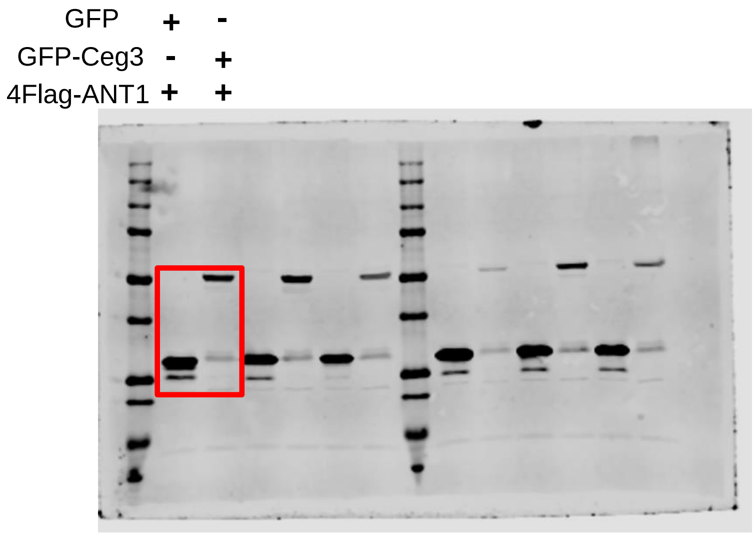

Input
IB:GFP

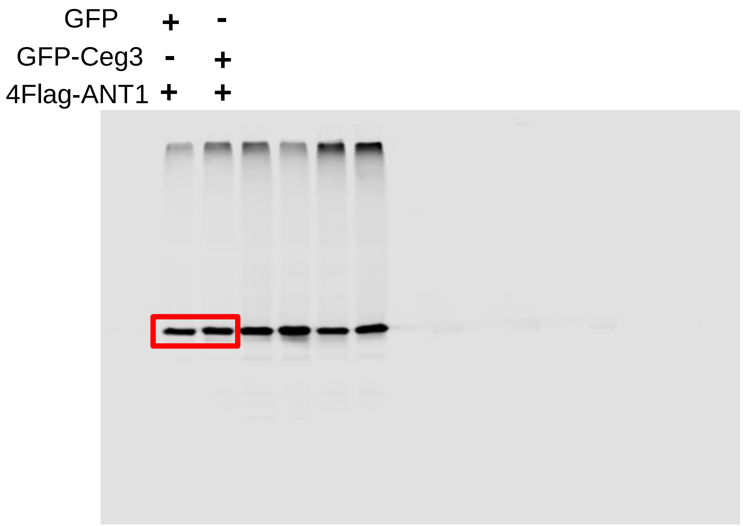

Input
IB:Flag

Figure 3A source data

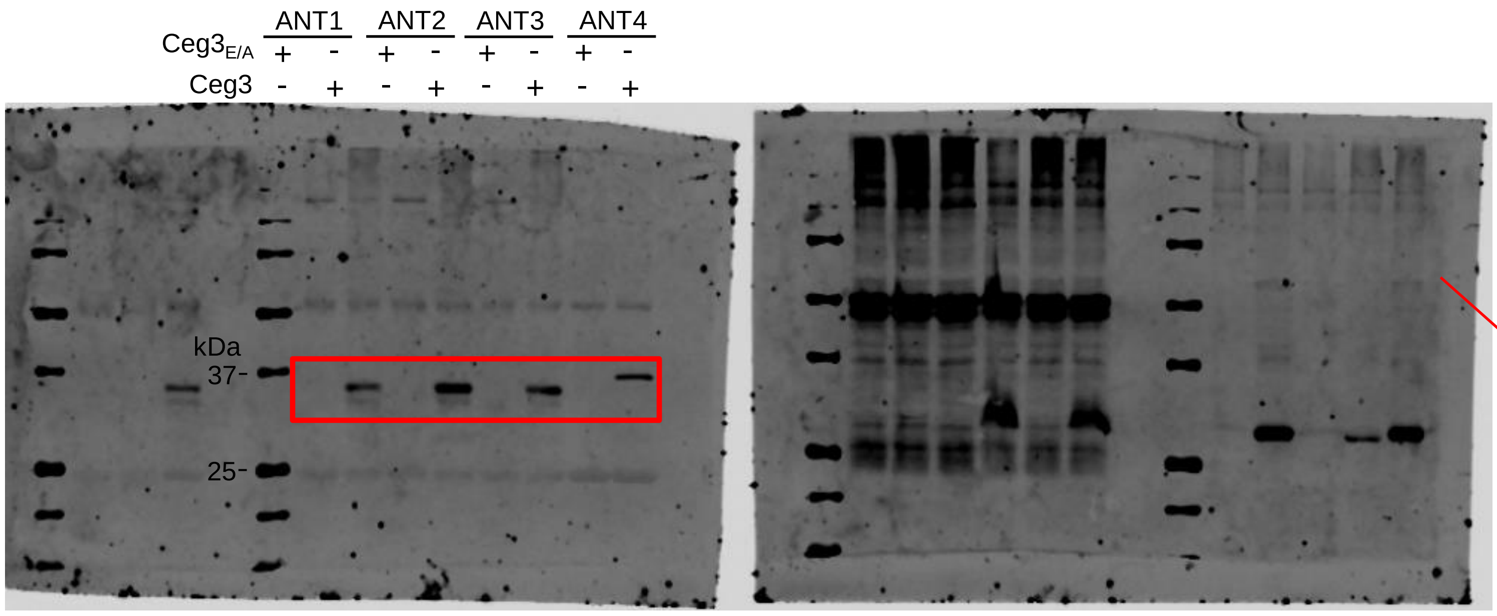

IB:Flag IB: ADPR

This membrane is for an unrelated experiment and was scanned simultaneously.

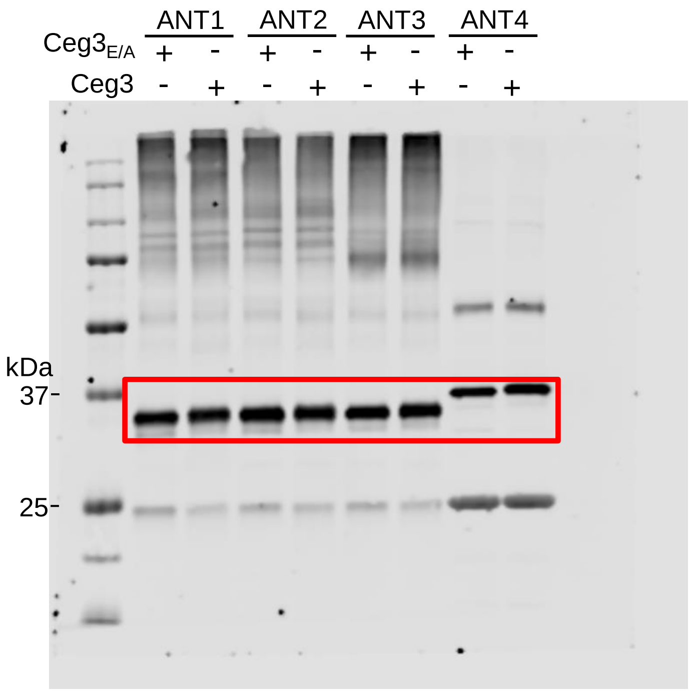

IB:Flag IB: Flag

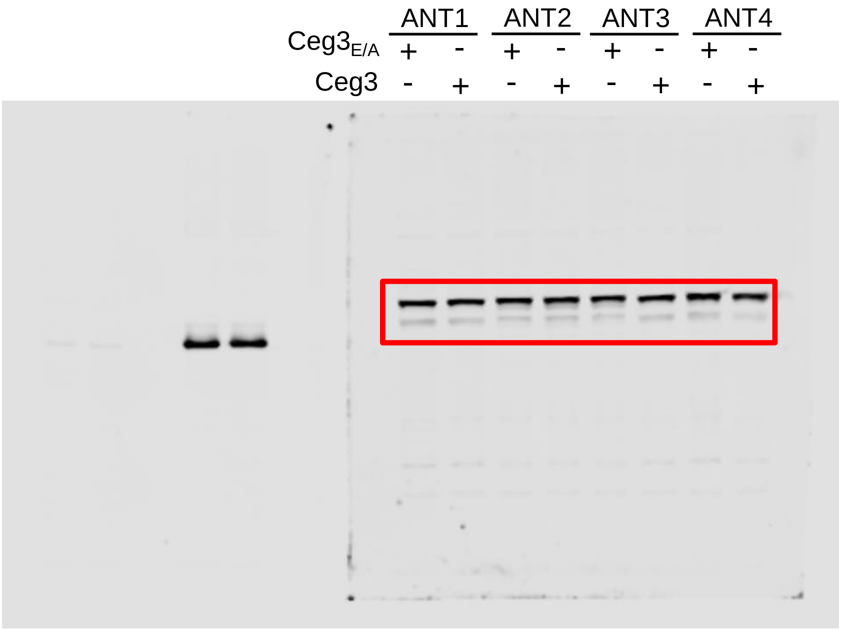

Input IB: GFP

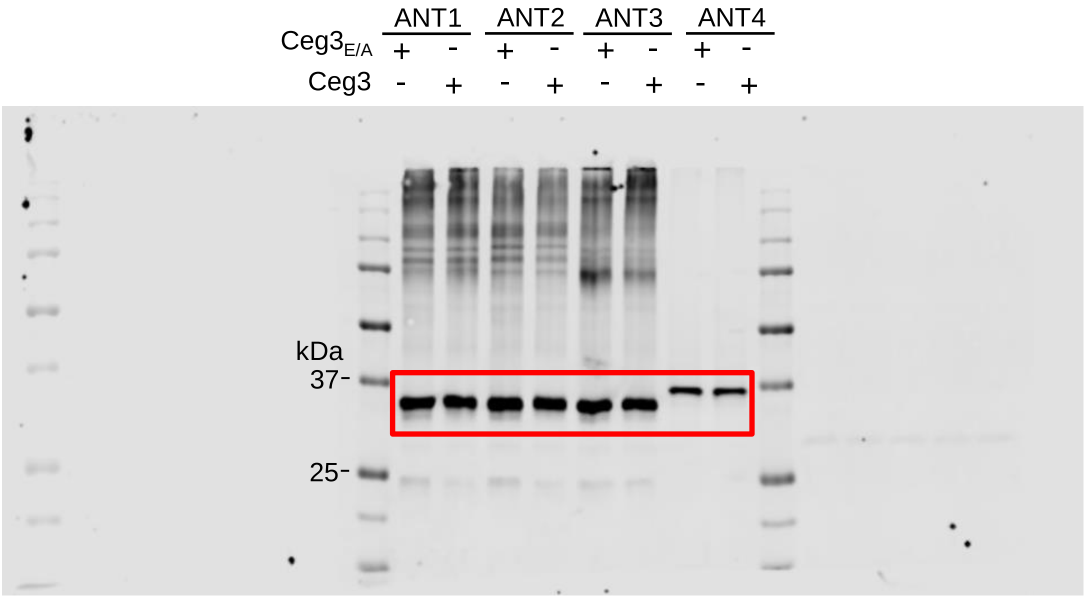

Input IB: Flag

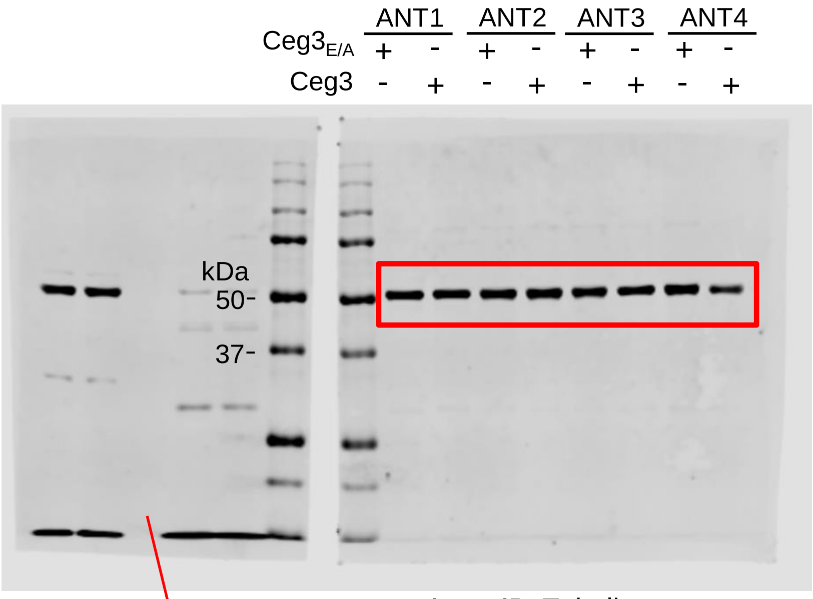

Input IB: Tubulin

This membrane is for an unrelated experiment and was scanned simultaneously.

Figure 3D source data

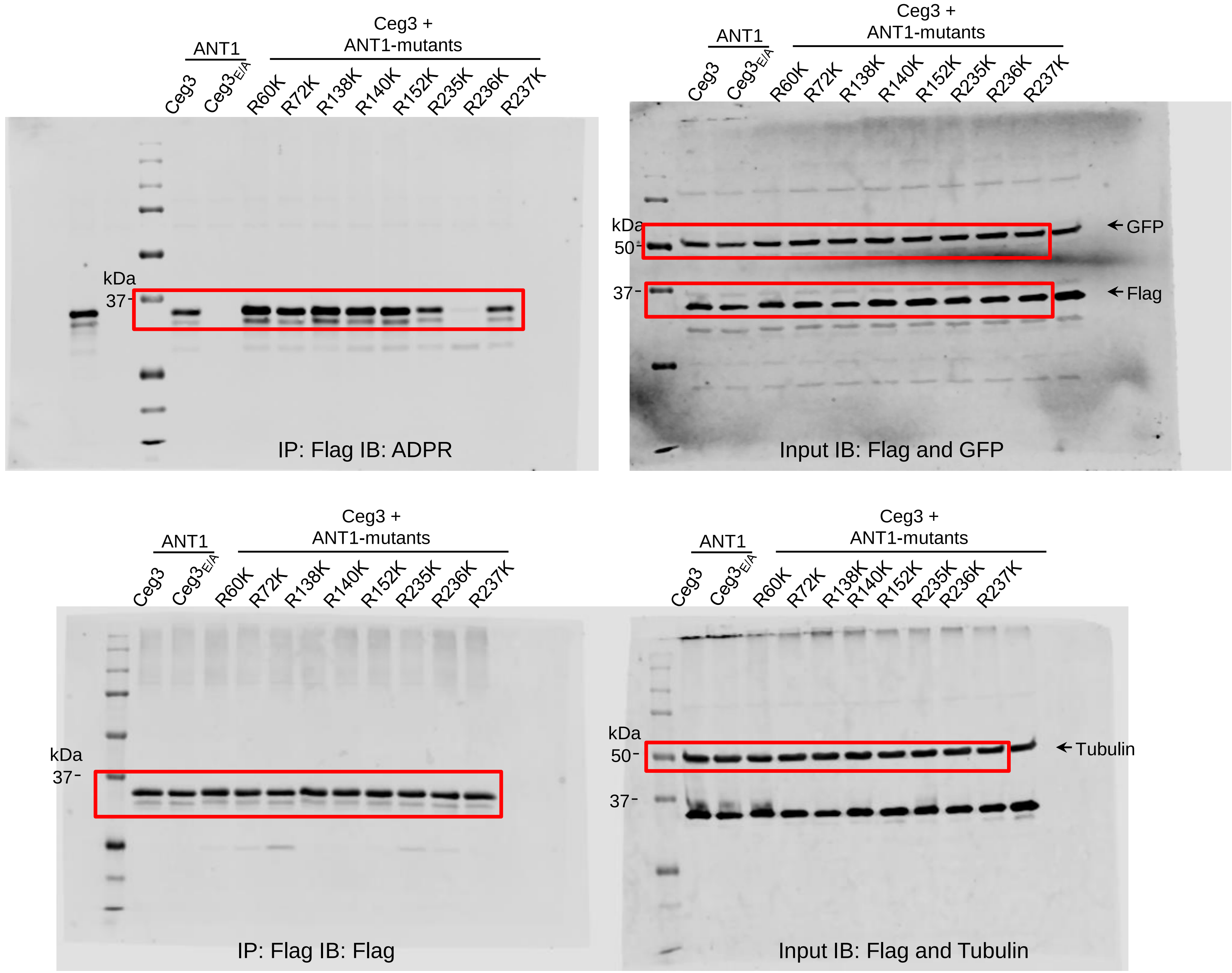

Figure 3E source data

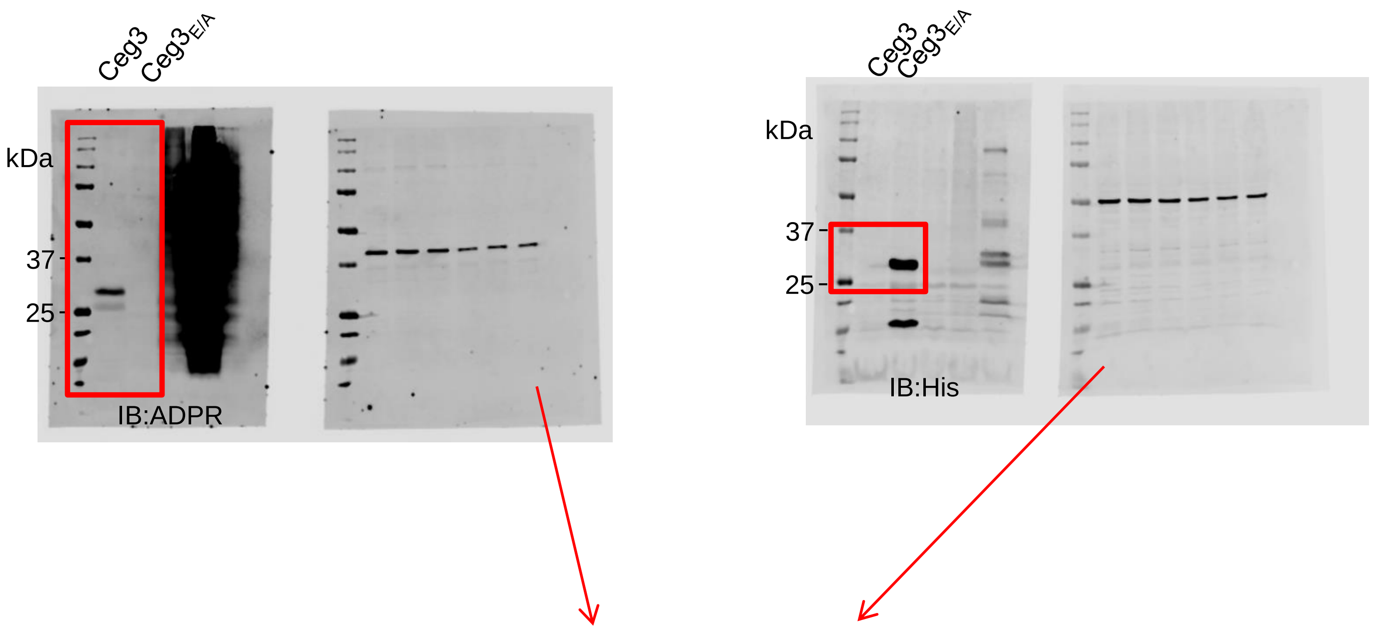

These membranes are for unrelated experiments and were scanned simultaneously.

Figure 4A source data

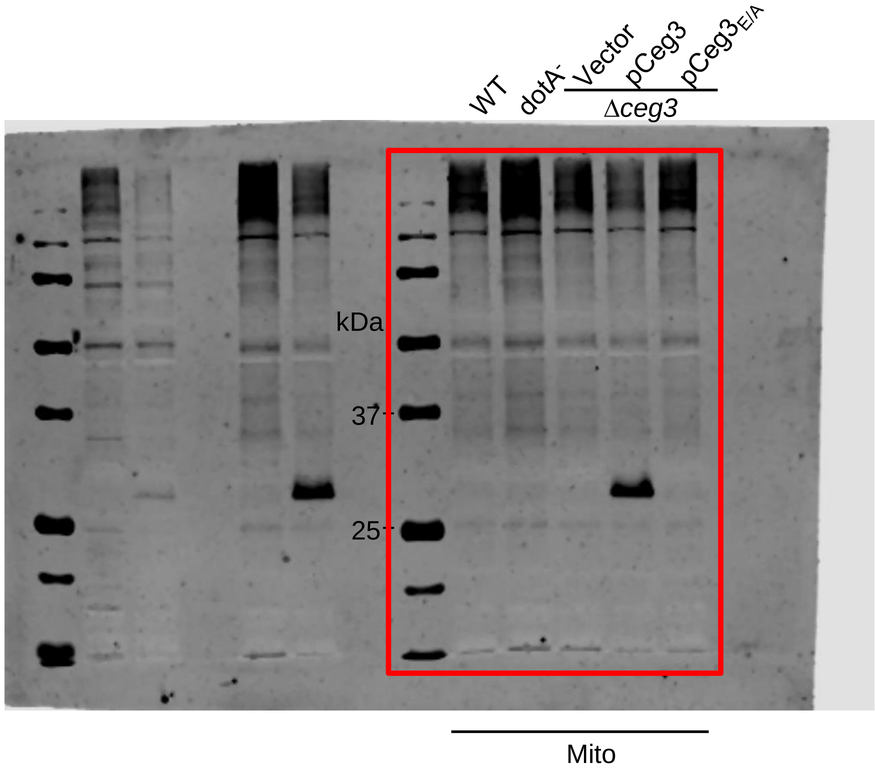

IB: ADPR

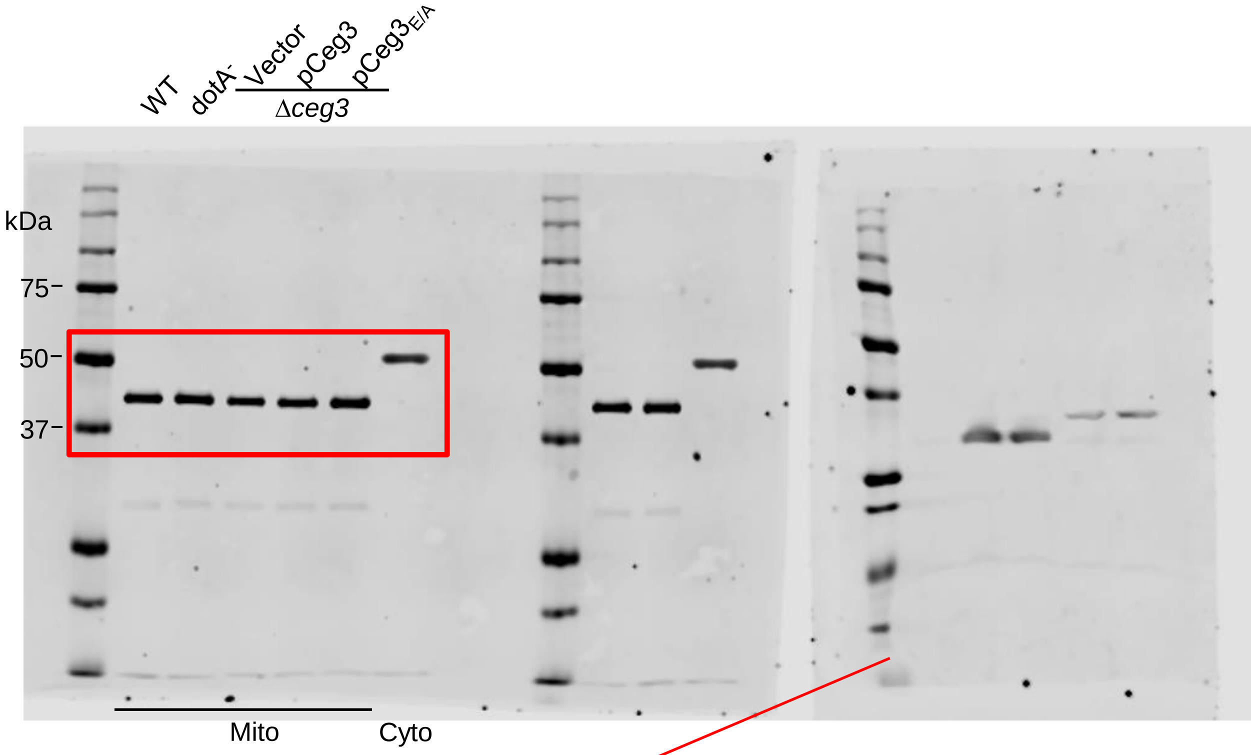

IB: PDHA1 and Tubulin

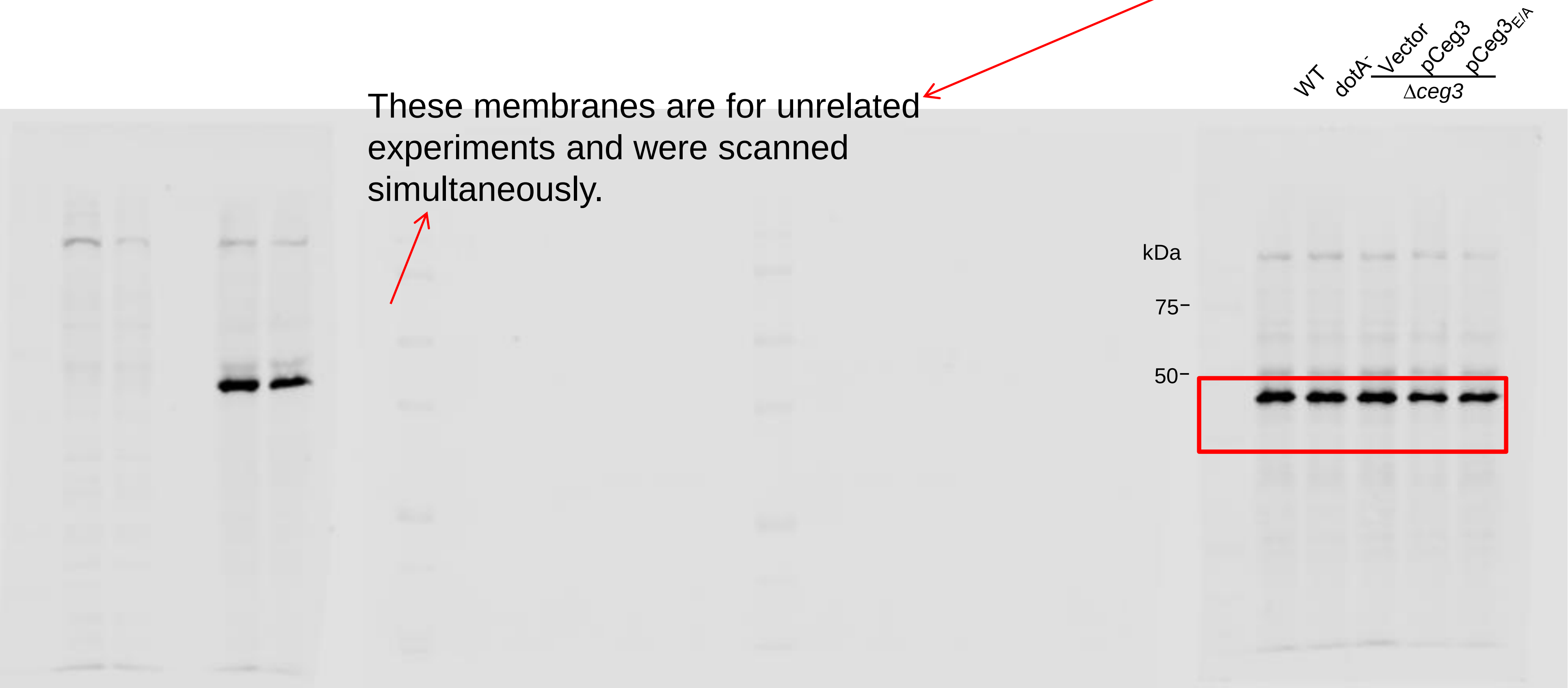

Expression

IB: ICDH

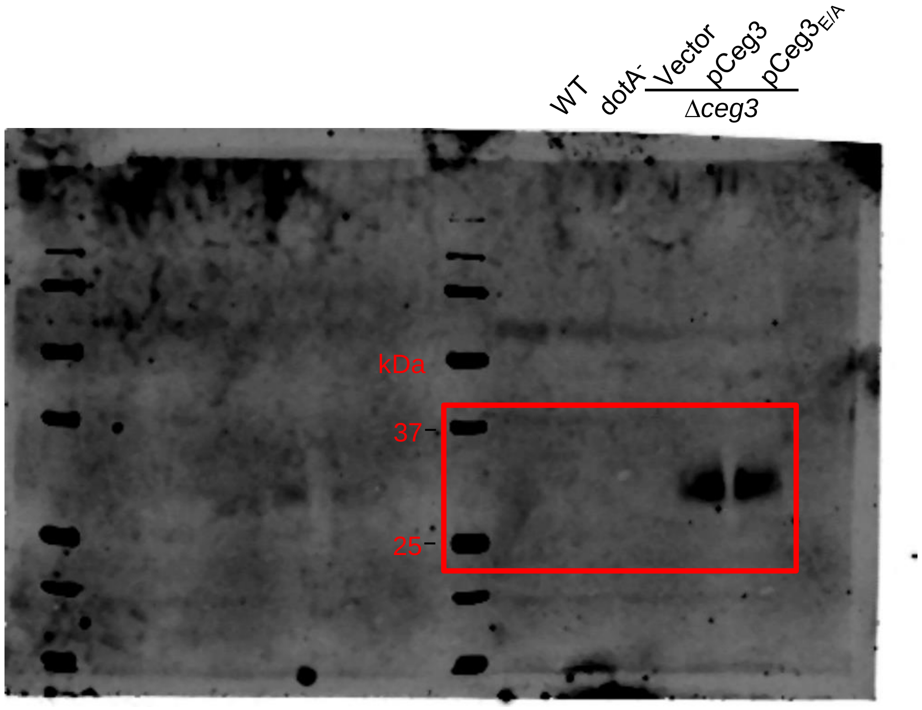

Expression

IB: Flag

Figure 4B source data

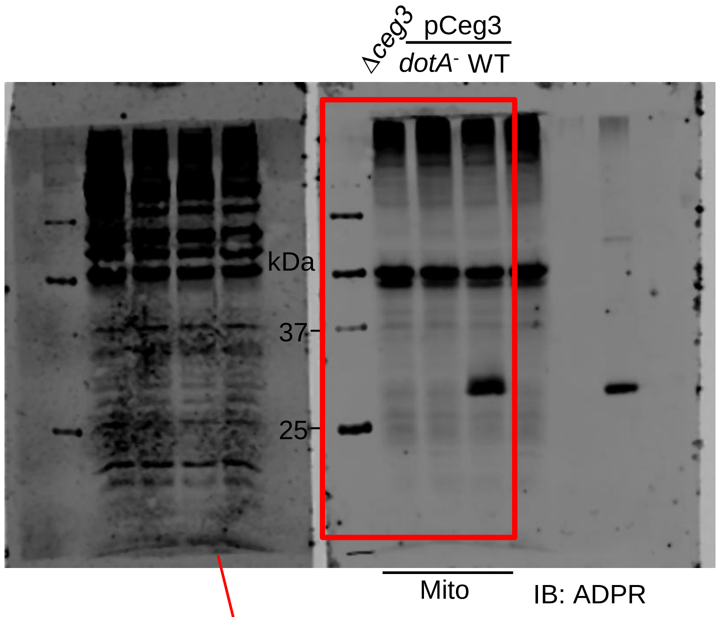

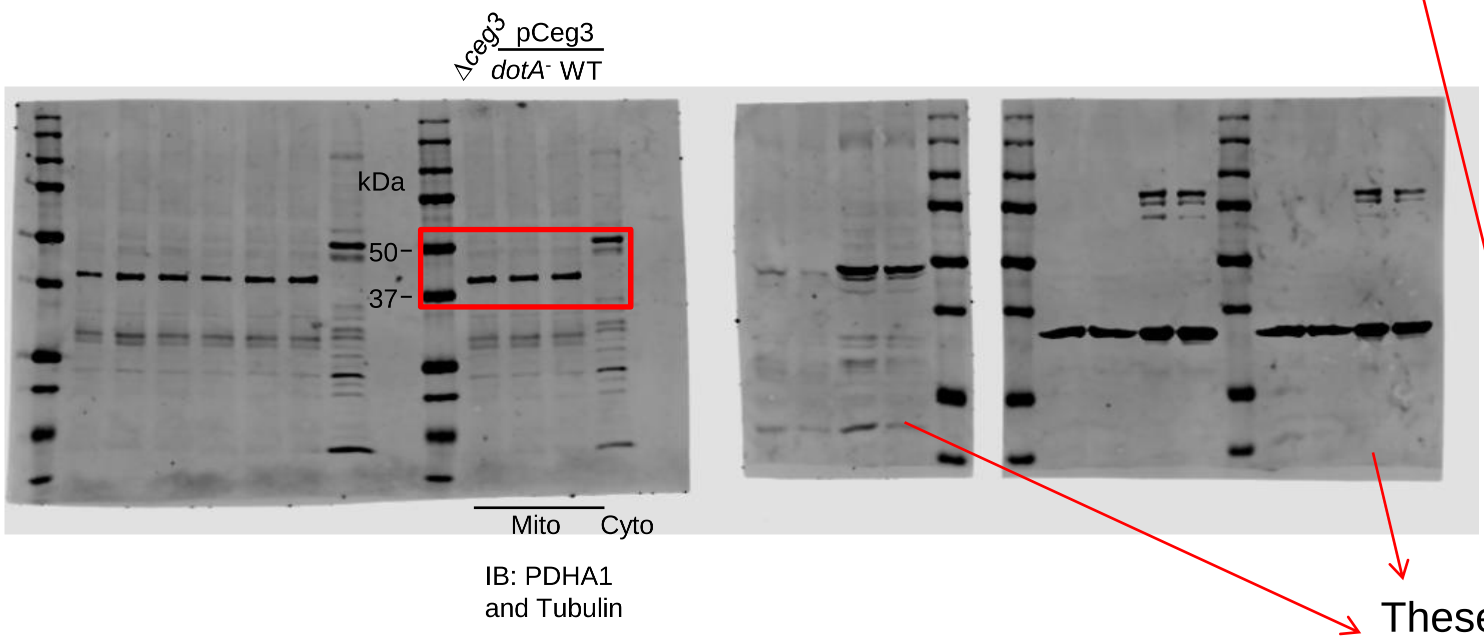

These membranes are for unrelated experiments and were scanned simultaneously.

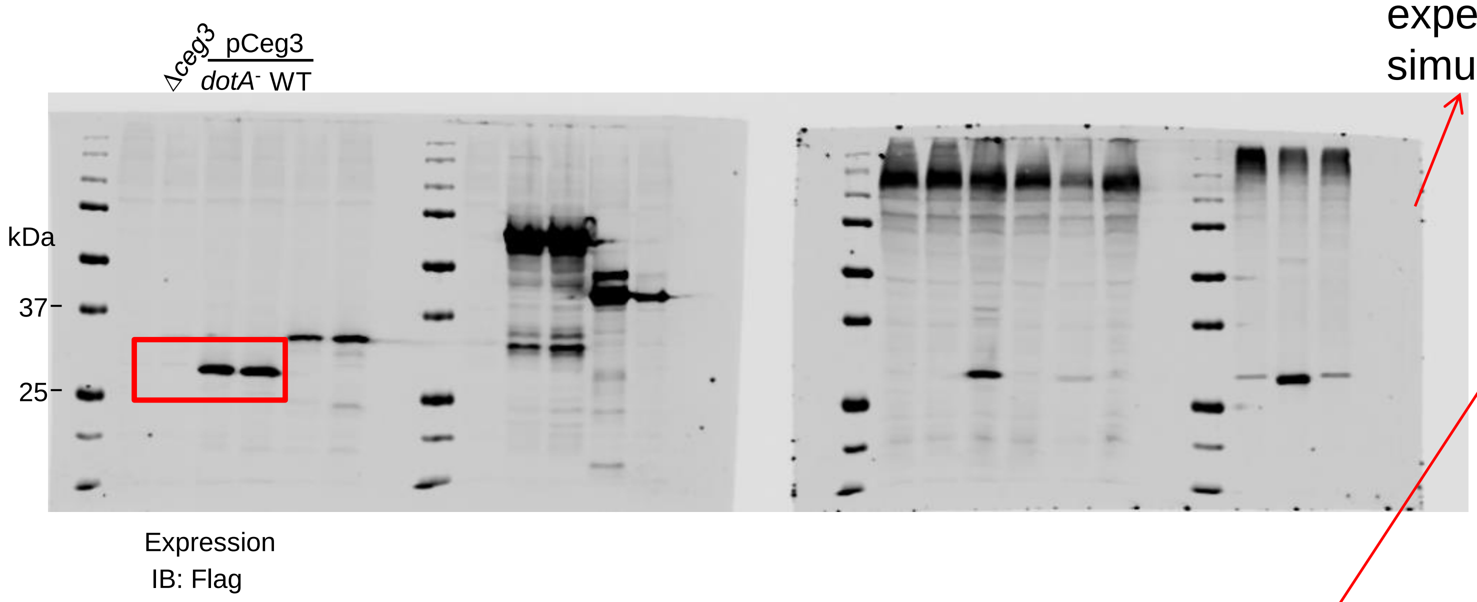

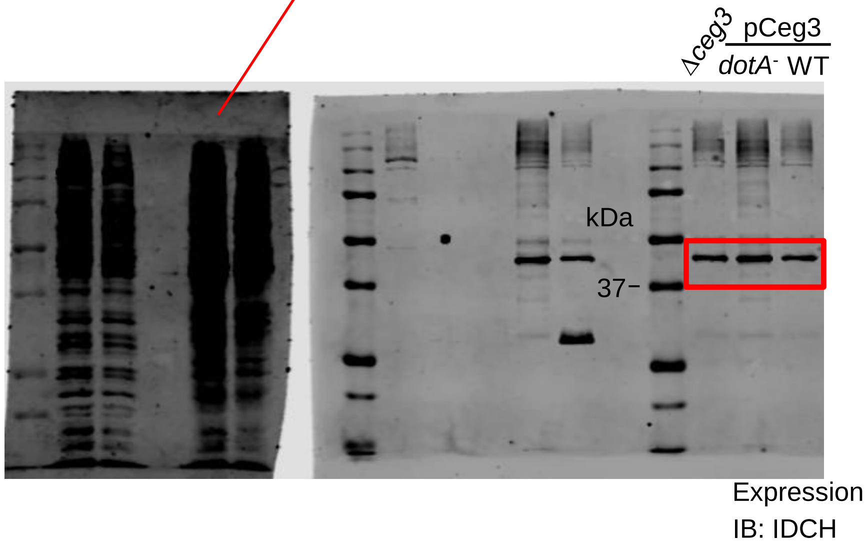

Figure 4C source data

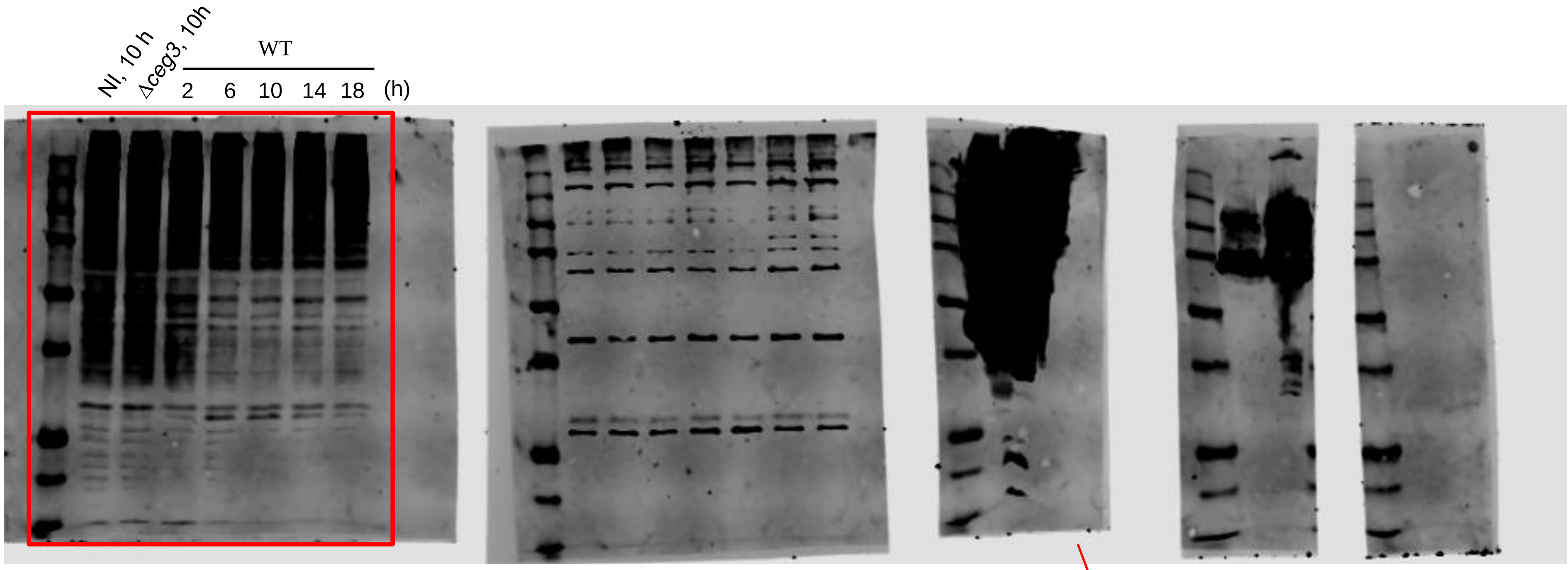

IB: ADPR

These membranes are for unrelated experiments and were scanned simultaneously.

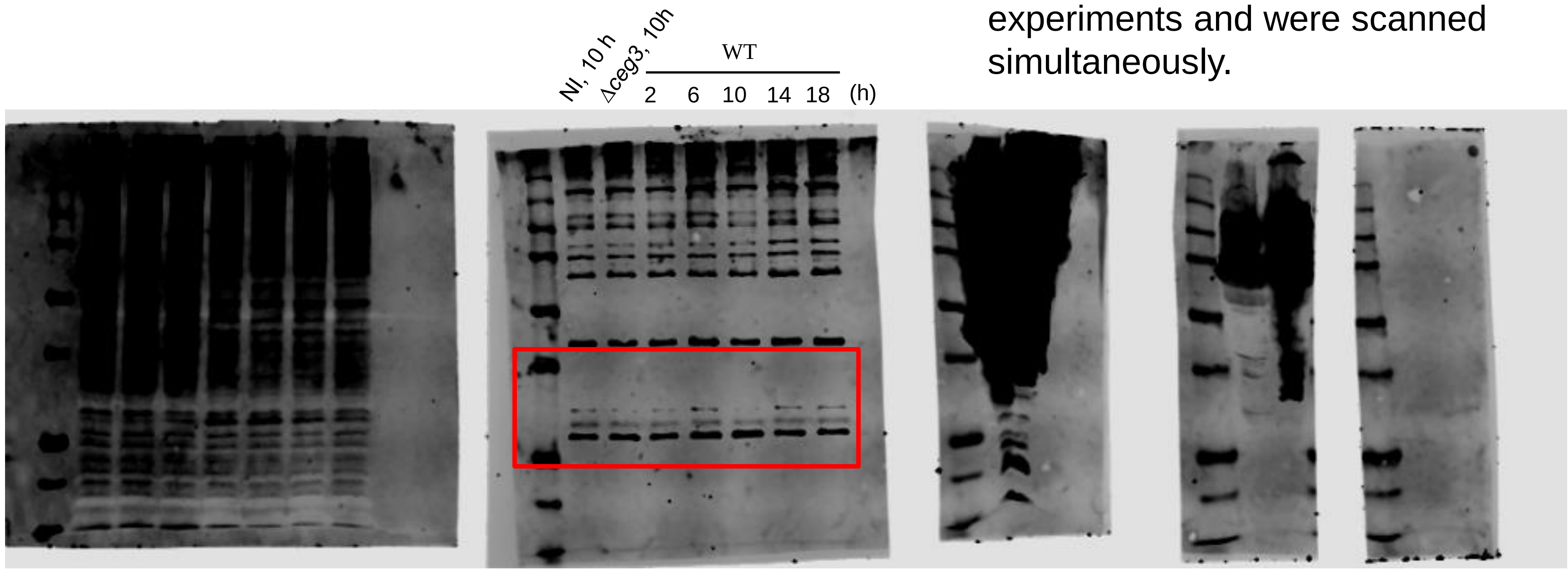

IB: ANTs

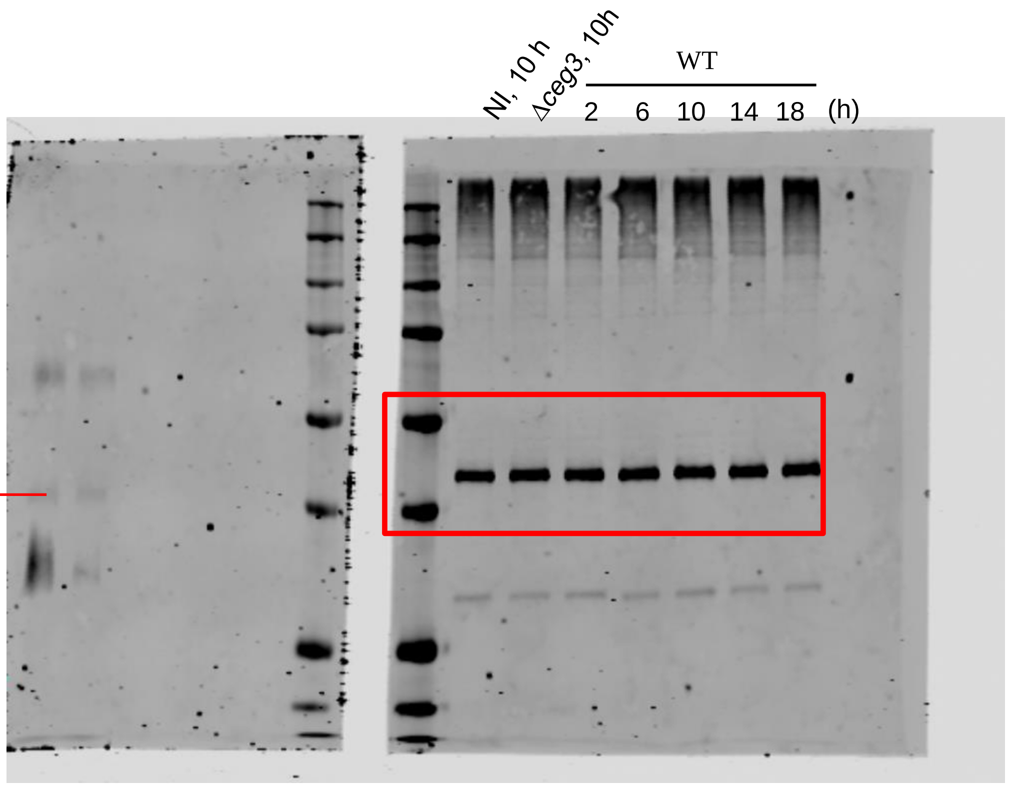

This membrane is for an unrelated experiment and was scanned simultaneously.

IB: PDHA1

Figure 3-figure supplement 1 source data

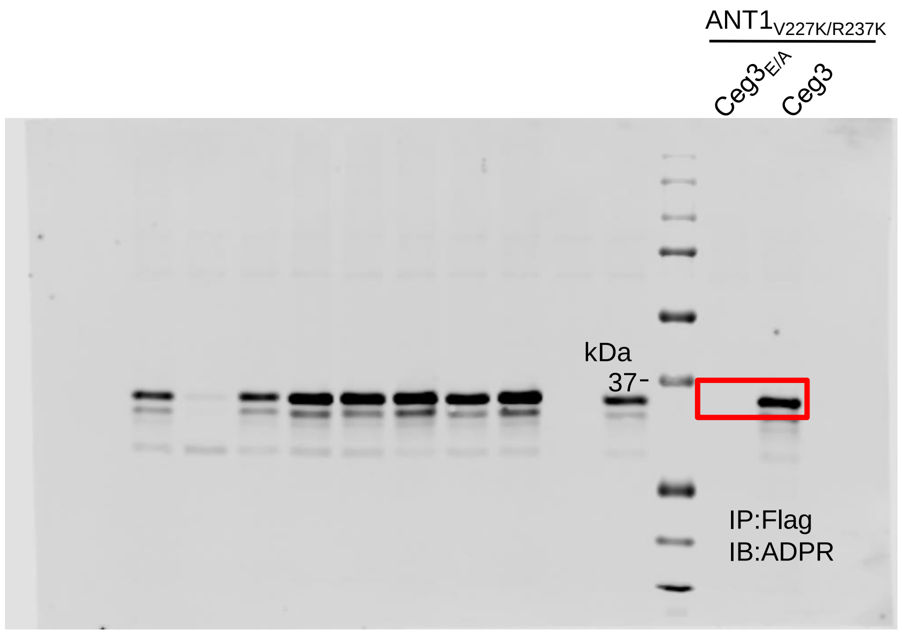

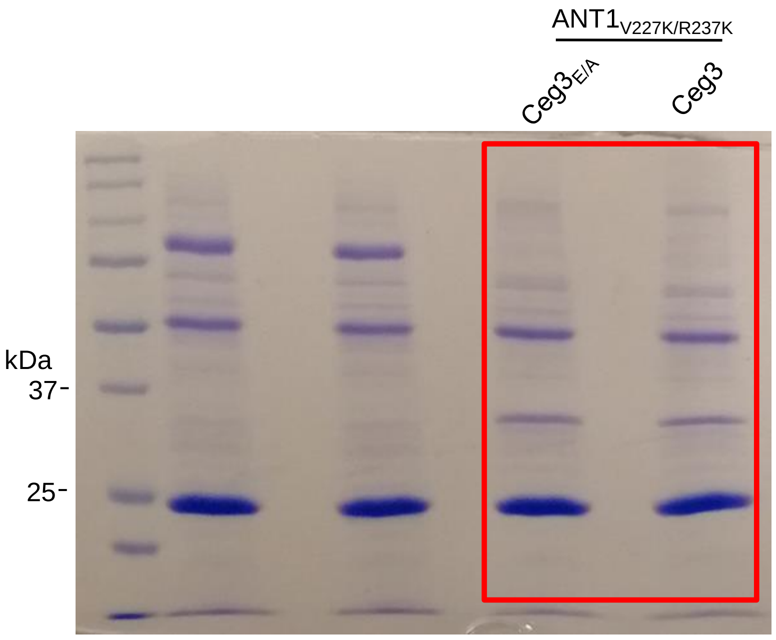

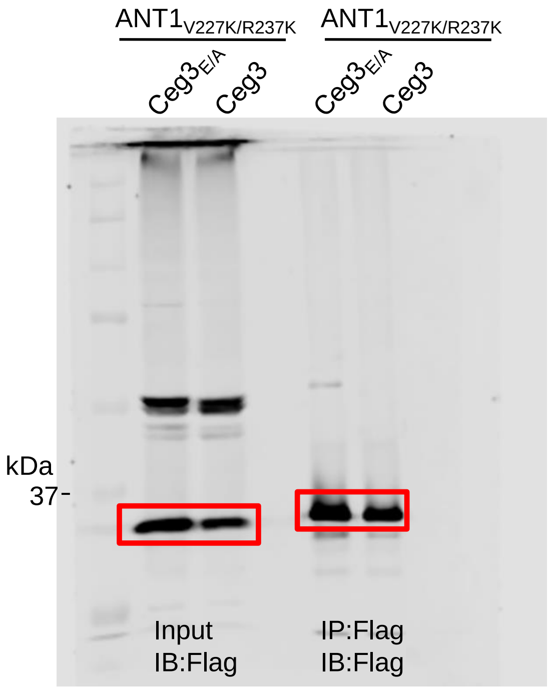

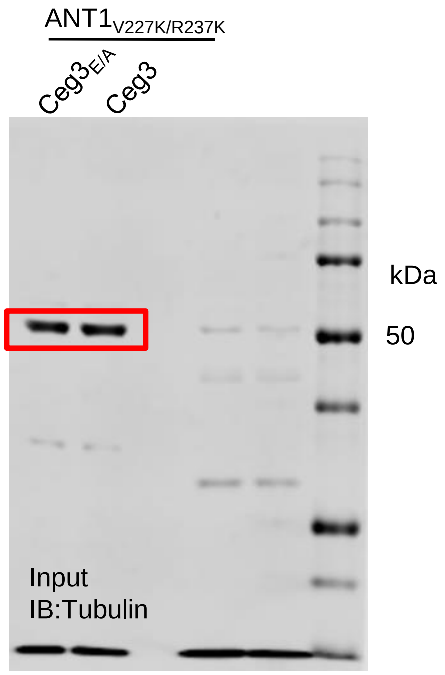

Figure 3-figure supplement 3 source data

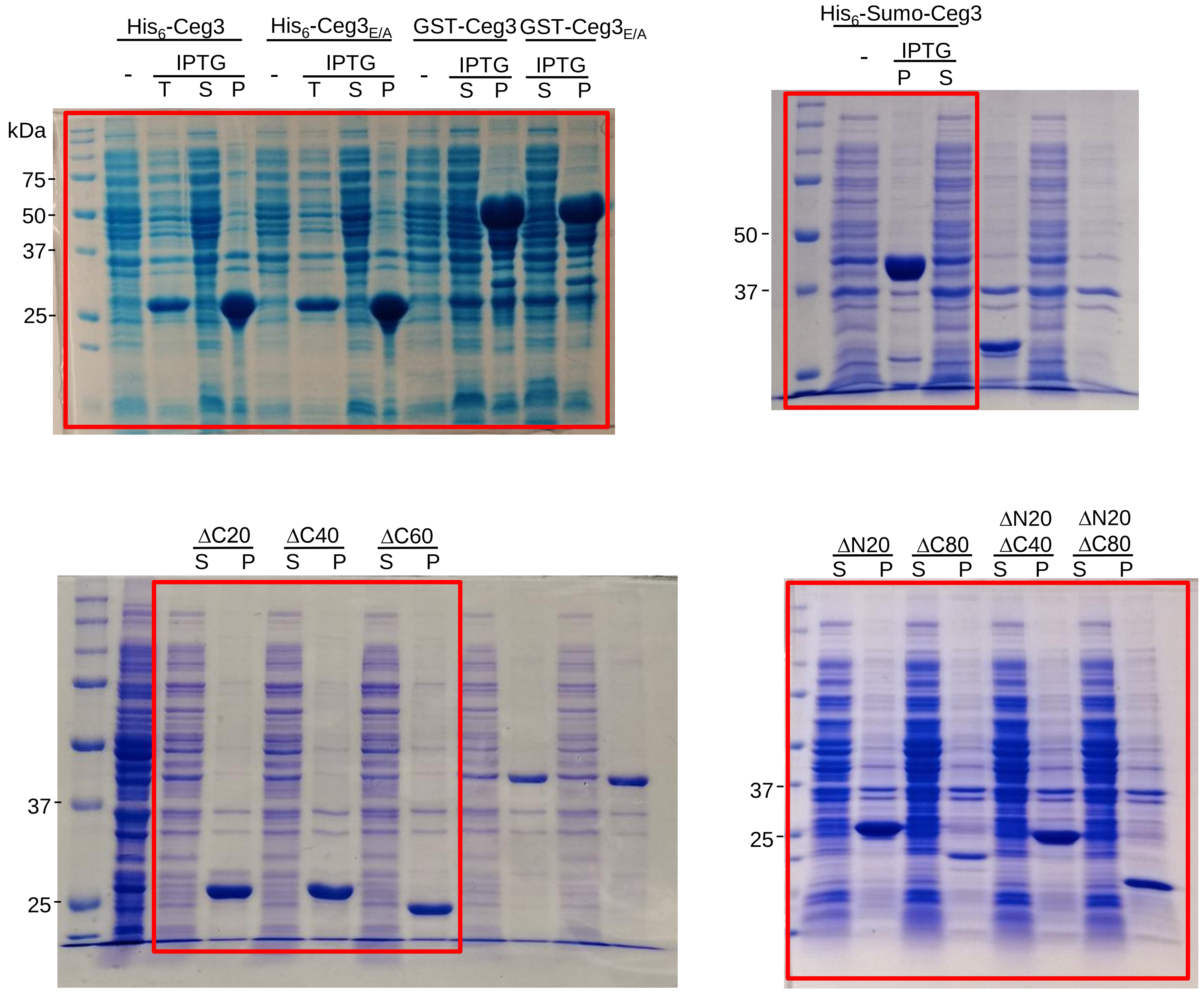

Figure 3-figure supplement 4 source data

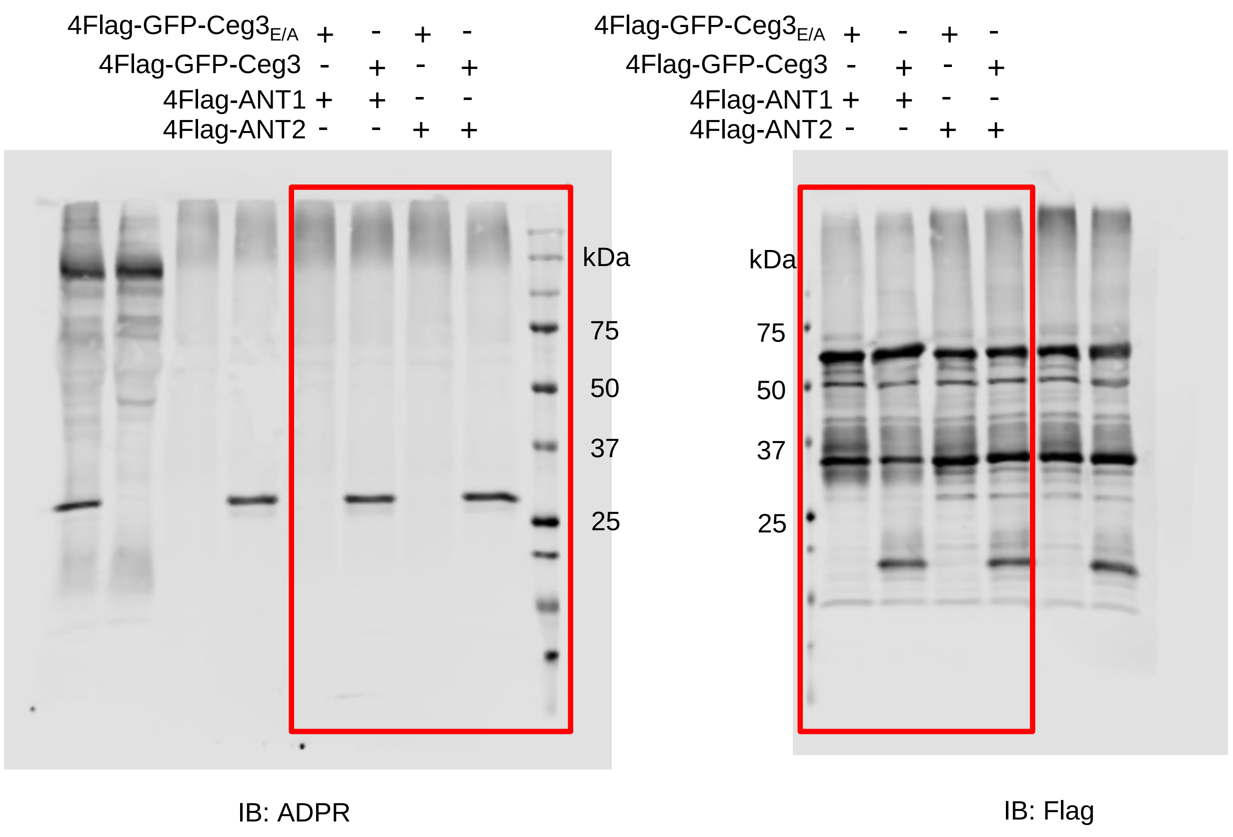

Figure 5-figure supplement 1A source data

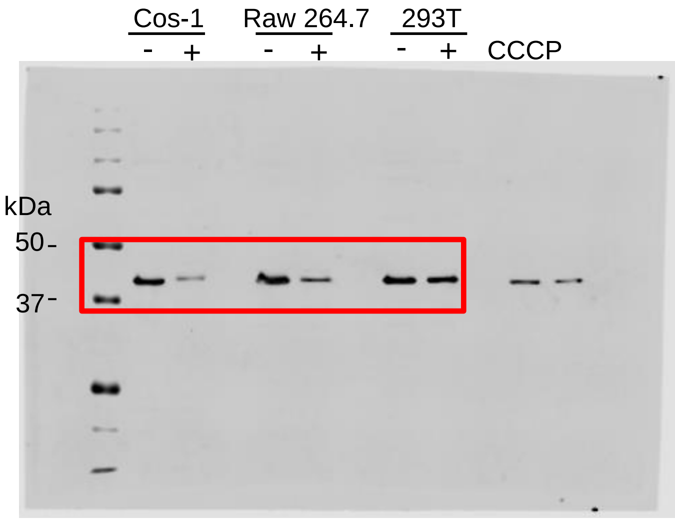

IB: PDHA1

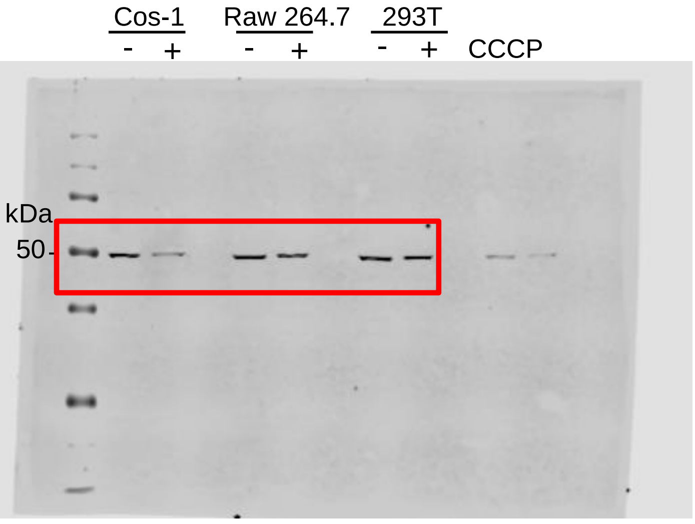

IB: ATPB

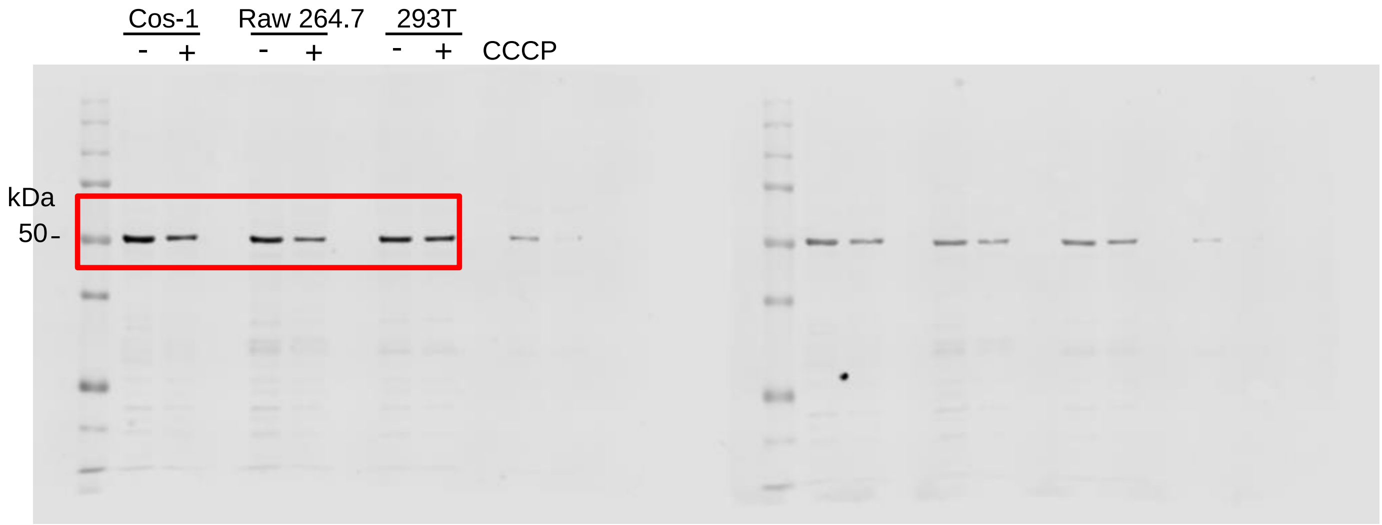

IB: Tubulin

Figure 5-figure supplement 1B source data

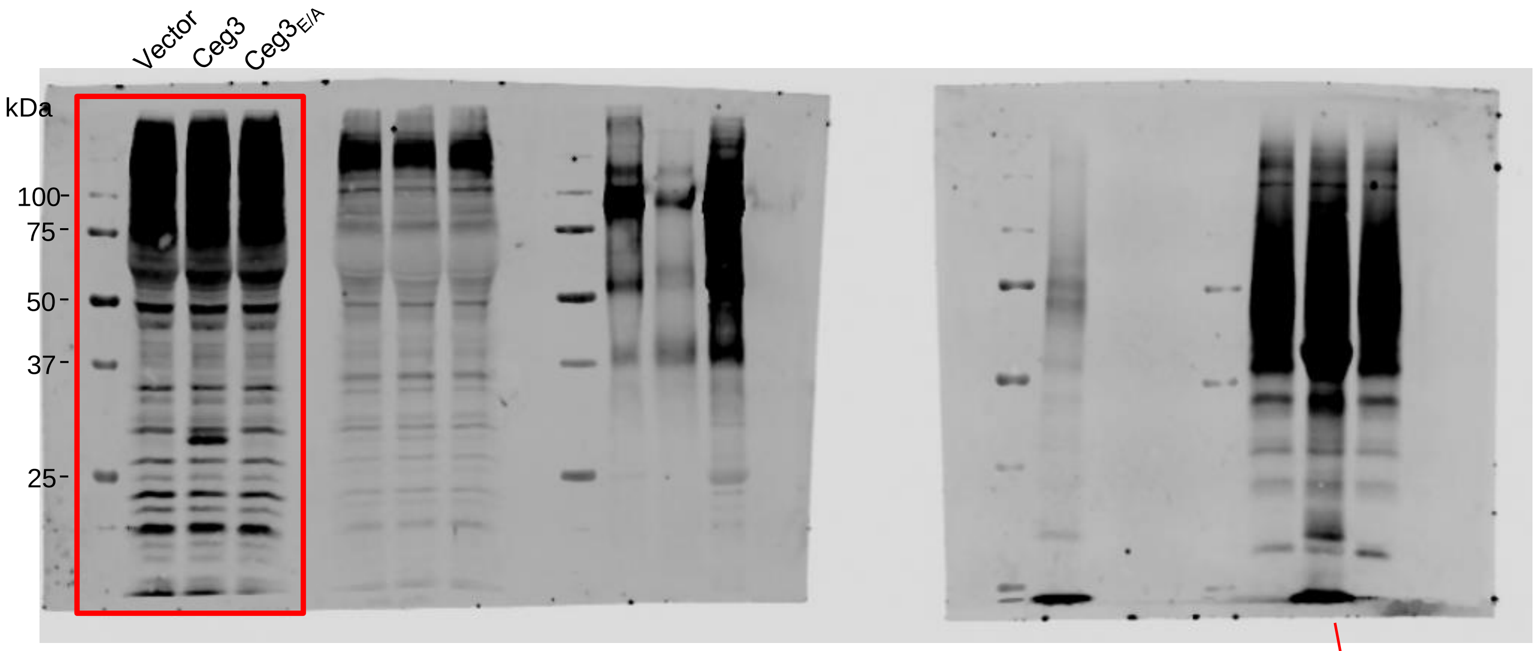

IB: ADPR

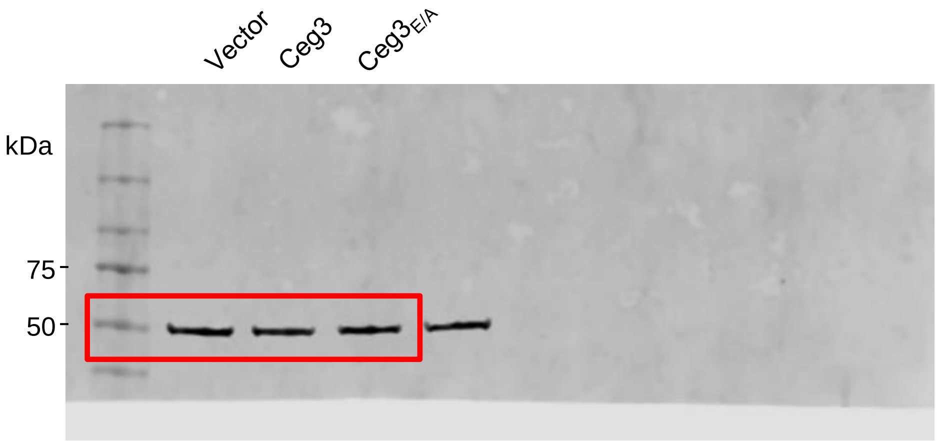

IB: Tubulin

This membrane is for an unrelated experiment and was scanned simultaneously.

Figure 5-figure supplement 1C source data

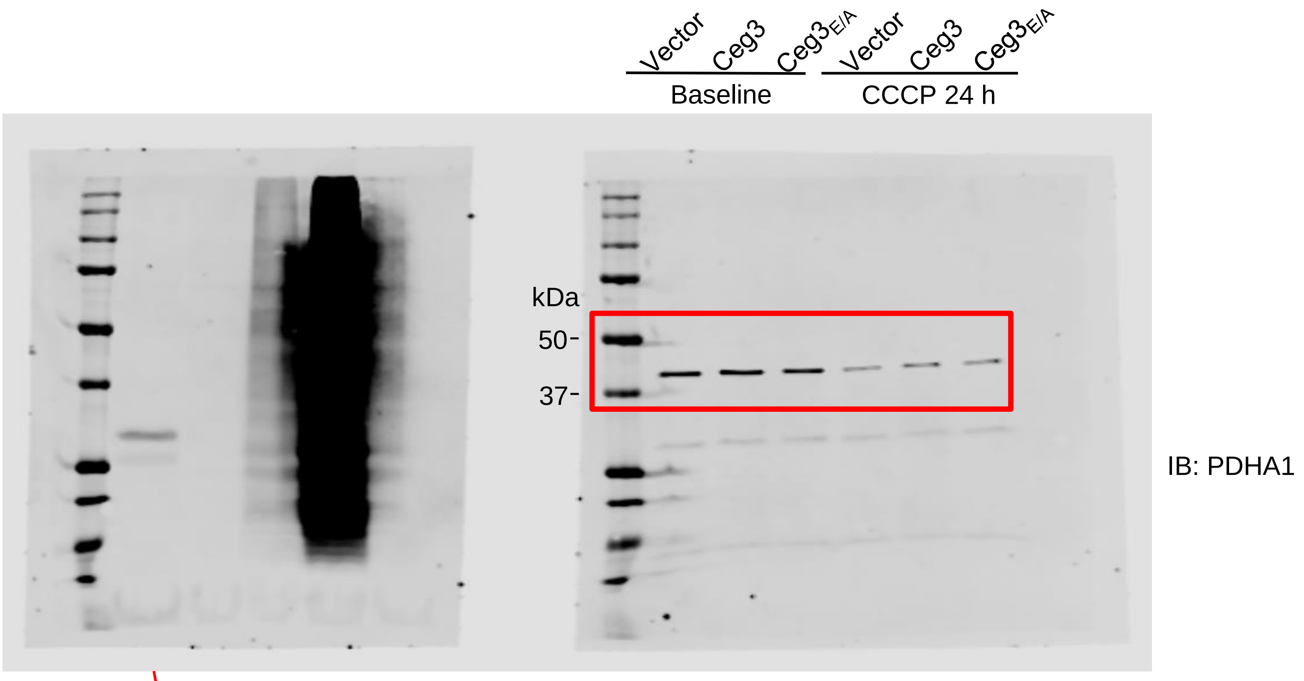

These membranes are for unrelated experiments and were scanned simultaneously.

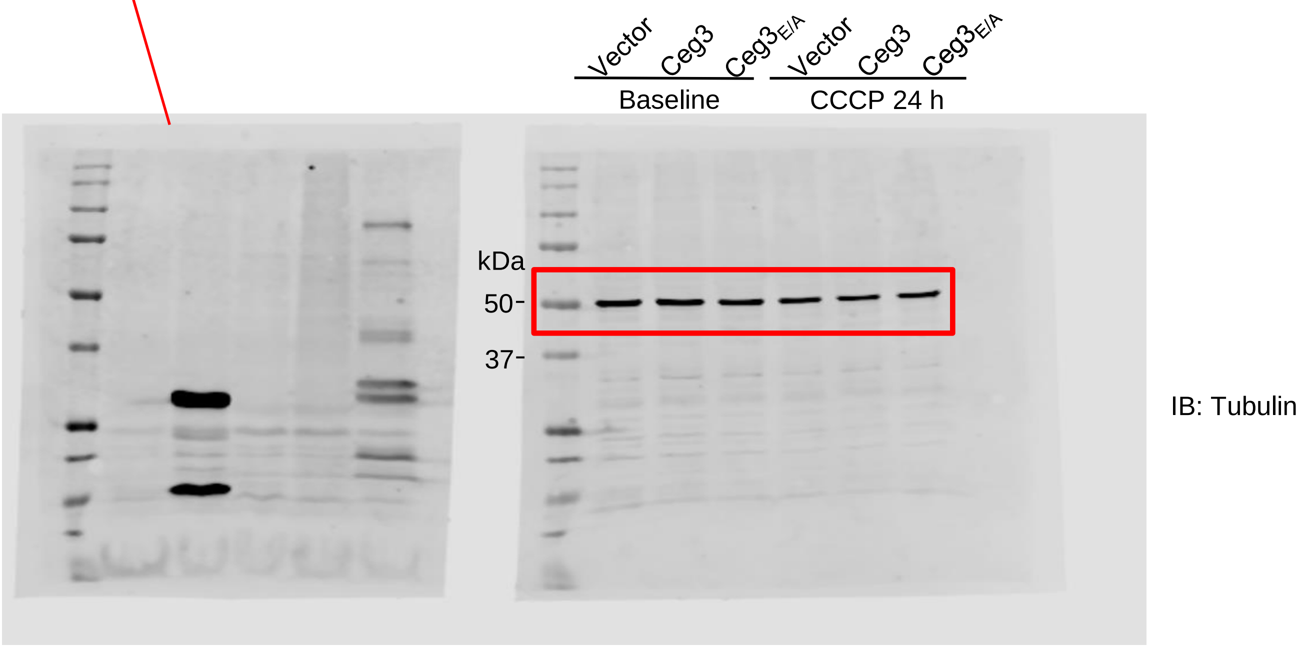

Supplement: Source data 1. [file elife-73611-data1.pdf]
