# Supplementary figures and images for: Legionella pneumophila modulates host energy metabolism by ADP-ribosylation of ADP/ATP translocases
Source: eLife. 2022 Jan 27;11:e73611. doi: 10.7554/eLife.73611 (PMC8820735; doi:10.7554/eLife.73611)

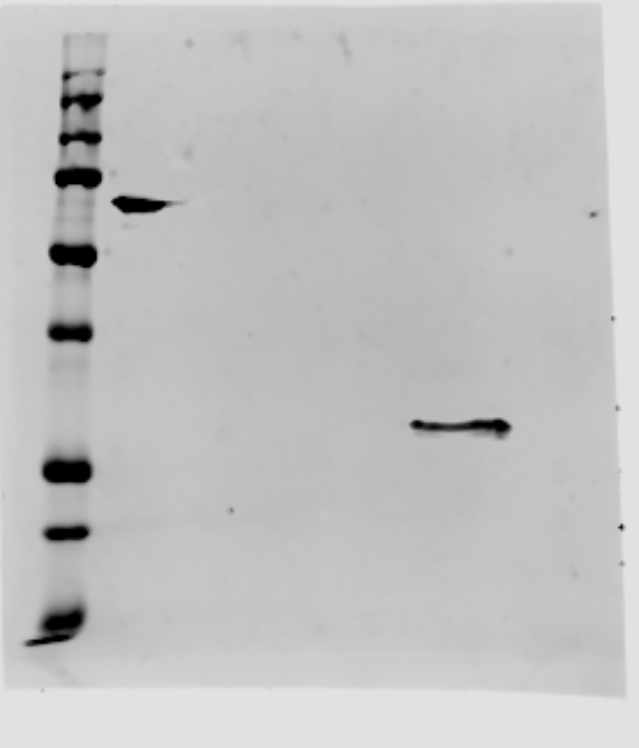

Supplement: Source data 2. [file elife-73611-data2.zip › raw original data/Figure 1 Source data raw panel E 1.tif]

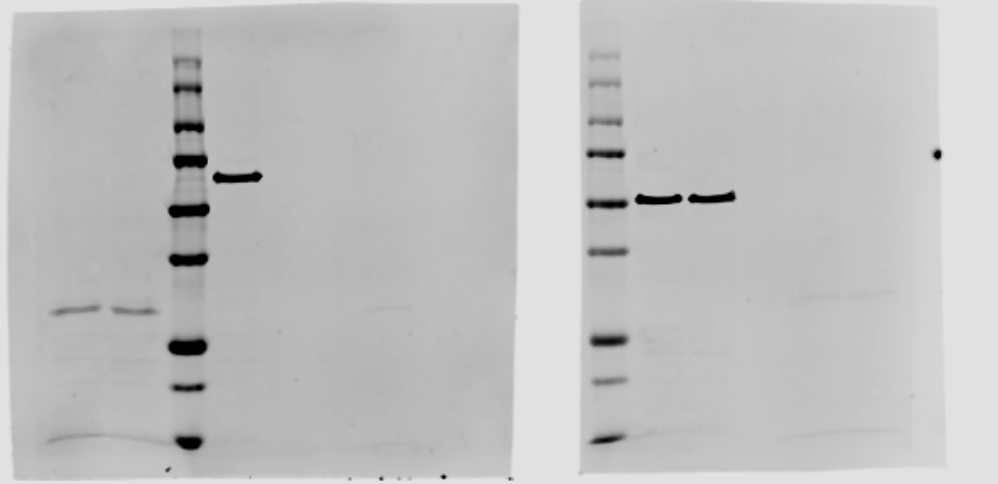

Supplement: Source data 2. [file elife-73611-data2.zip › raw original data/Figure 1 Source data raw panel E 2.tif]

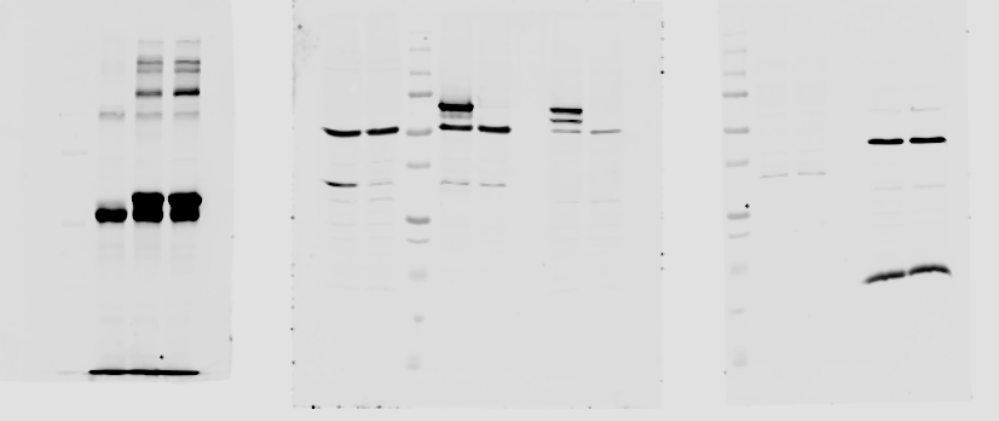

Supplement: Source data 2. [file elife-73611-data2.zip › raw original data/Figure 1 Source data raw panel E 3.tif]

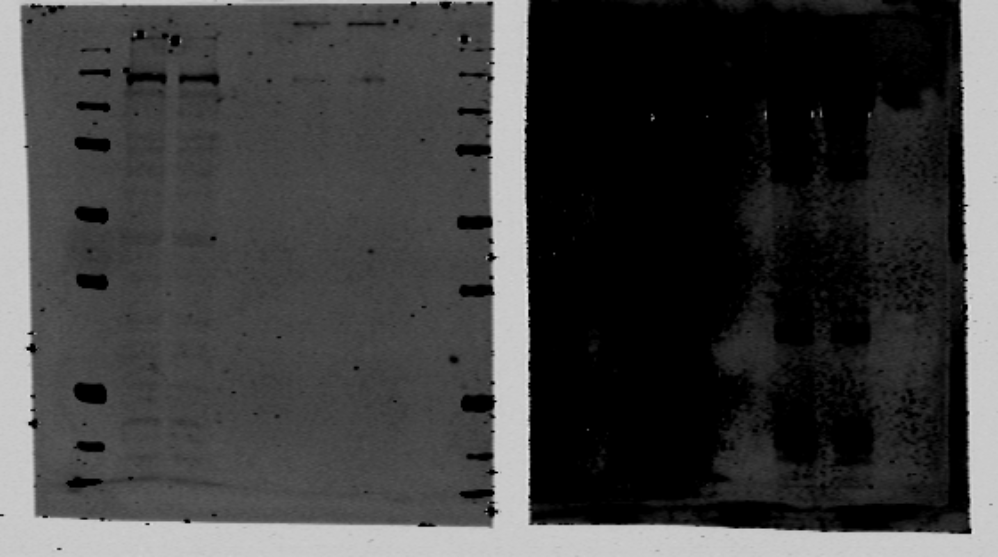

Supplement: Source data 2. [file elife-73611-data2.zip › raw original data/Figure 1 Source data raw panel E 4.tif]

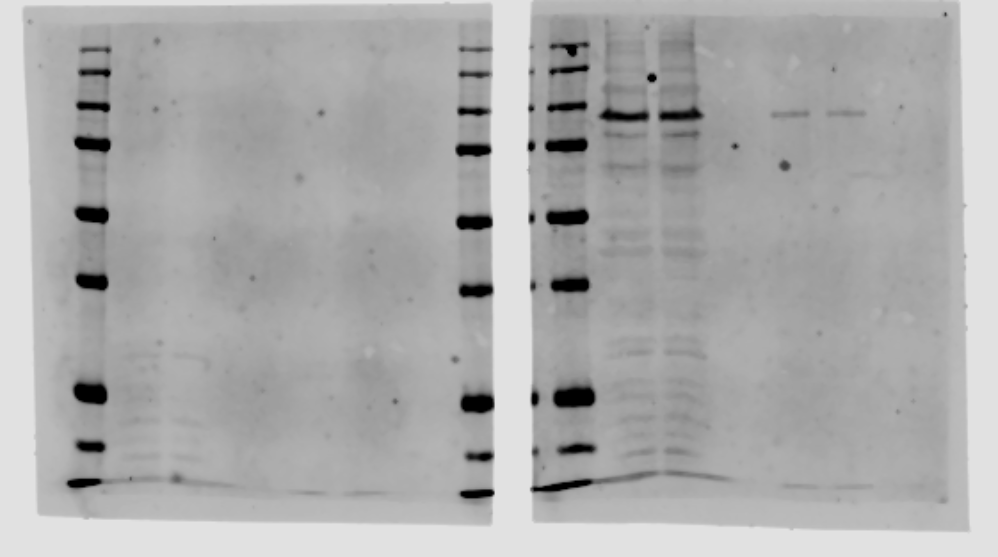

Supplement: Source data 2. [file elife-73611-data2.zip › raw original data/Figure 1 Source data raw panel E 5.tif]

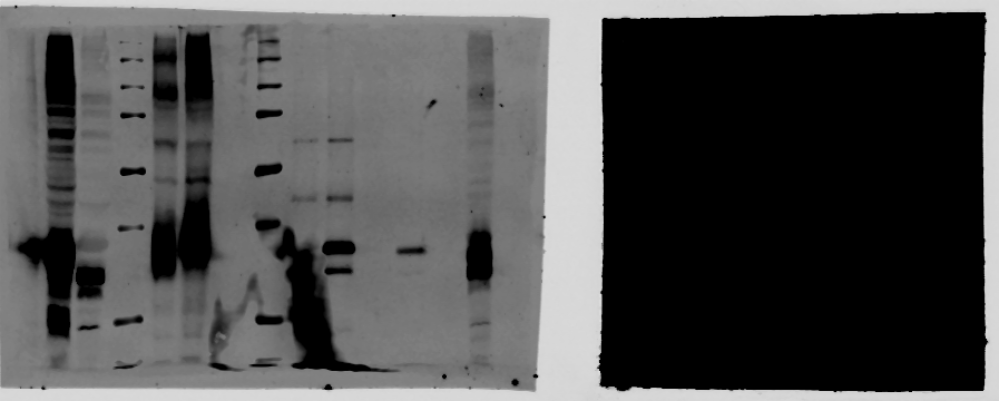

Supplement: Source data 2. [file elife-73611-data2.zip › raw original data/Figure 1 Source data raw panel F 1.tif]

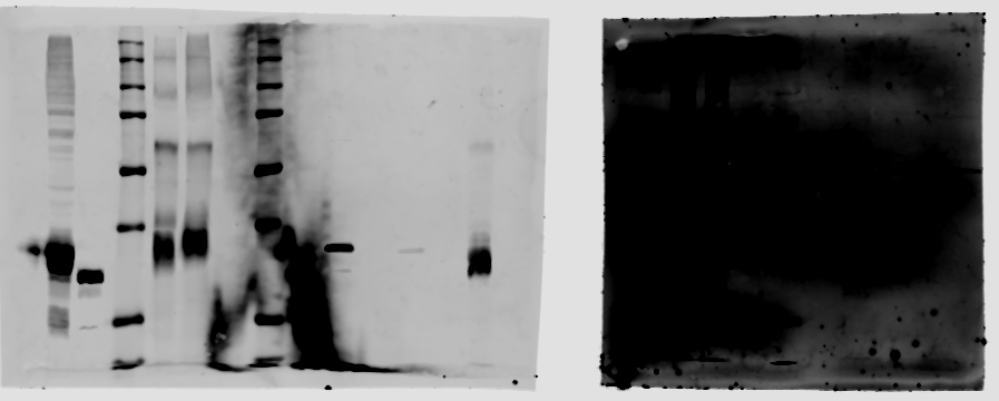

Supplement: Source data 2. [file elife-73611-data2.zip › raw original data/Figure 1 Source data raw panel F 2.tif]

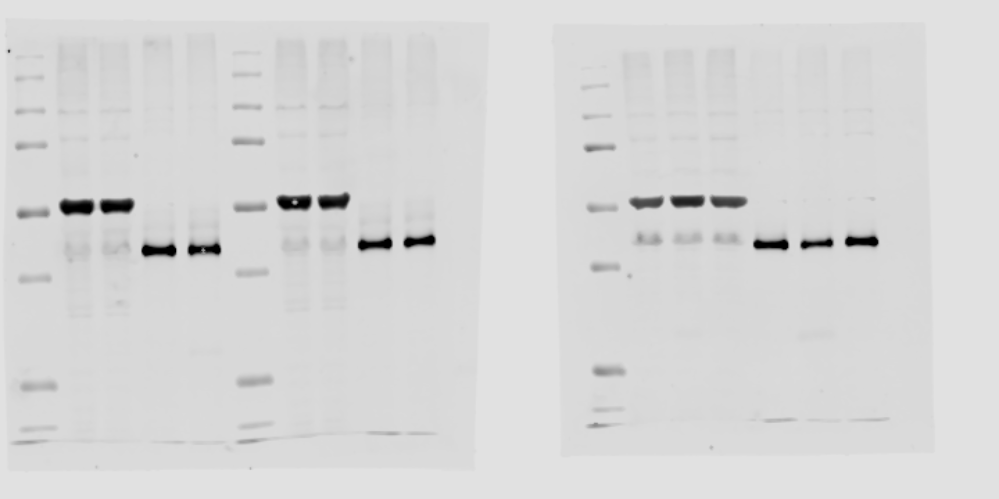

Supplement: Source data 2. [file elife-73611-data2.zip › raw original data/Figure 1 Source data raw panel F 3.tif]

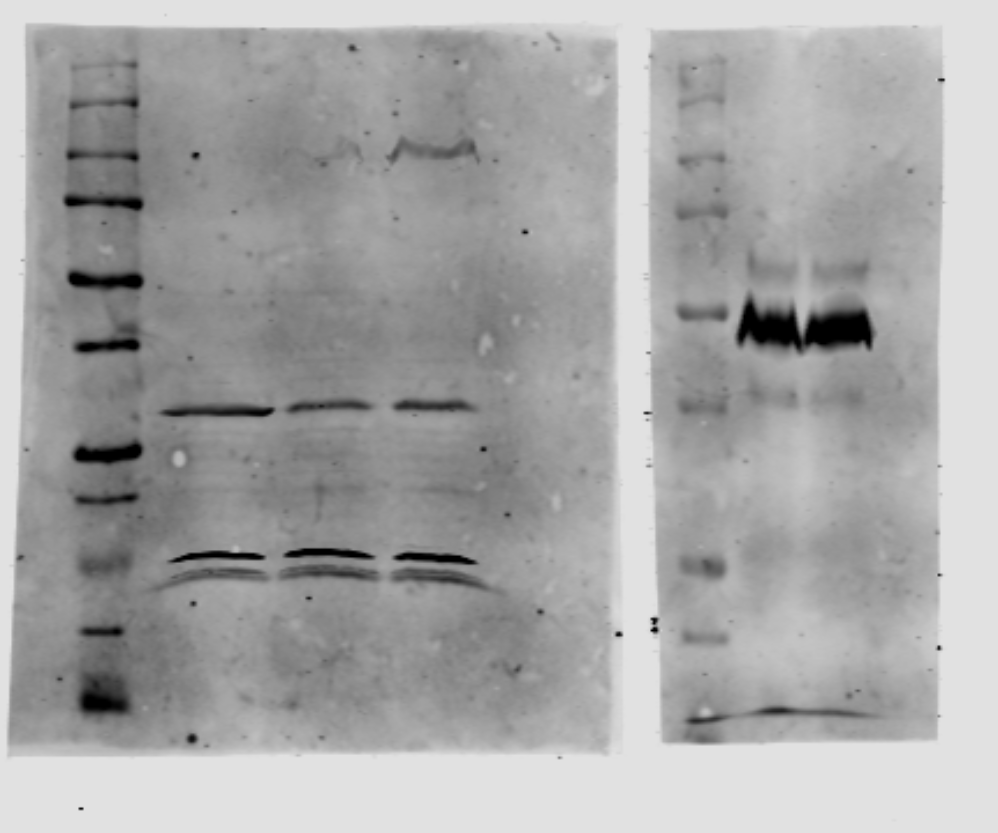

Supplement: Source data 2. [file elife-73611-data2.zip › raw original data/Figure 1 Source data raw panel F 4.tif]

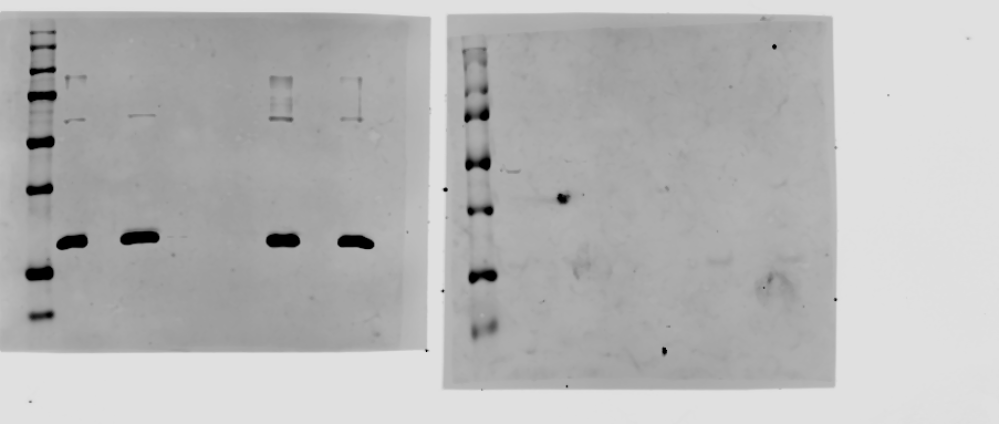

Supplement: Source data 2. [file elife-73611-data2.zip › raw original data/Figure 1 Source data raw panel G 1.tif]

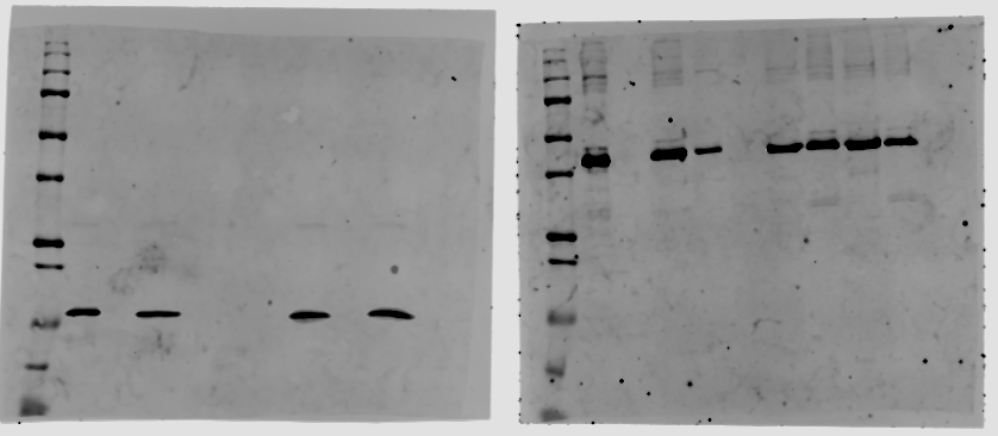

Supplement: Source data 2. [file elife-73611-data2.zip › raw original data/Figure 1 Source data raw panel G 2.tif]

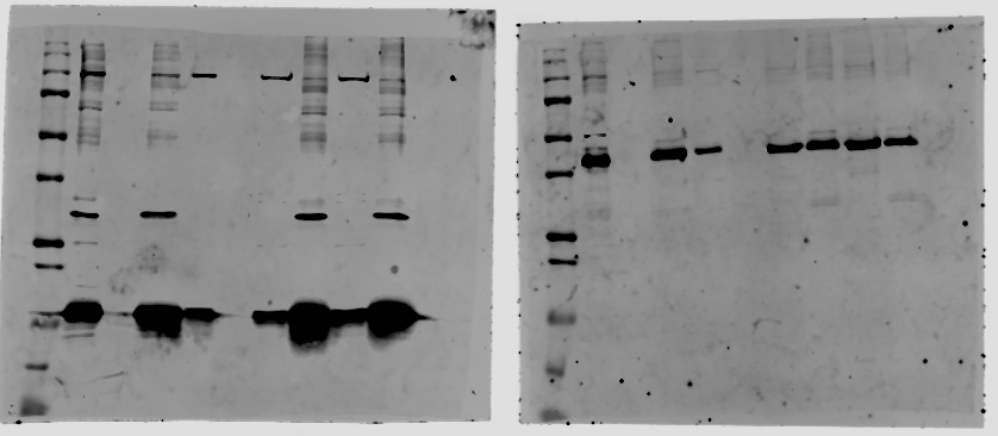

Supplement: Source data 2. [file elife-73611-data2.zip › raw original data/Figure 1 Source data raw panel G 3.tif]

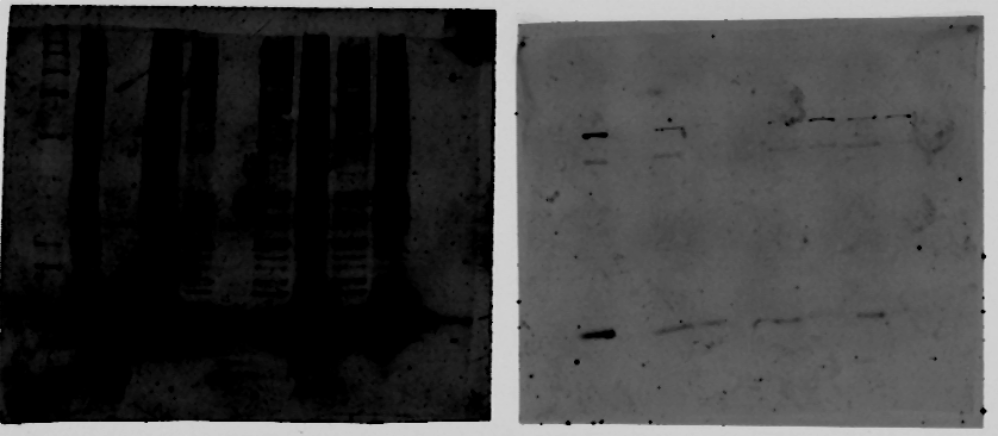

Supplement: Source data 2. [file elife-73611-data2.zip › raw original data/Figure 1 Source data raw panel G 4.tif]

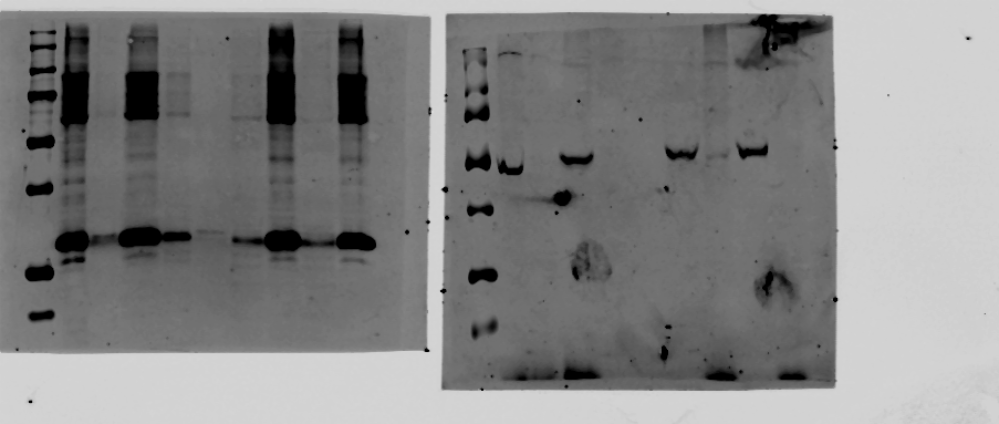

Supplement: Source data 2. [file elife-73611-data2.zip › raw original data/Figure 1 Source data raw panel G 5.tif]

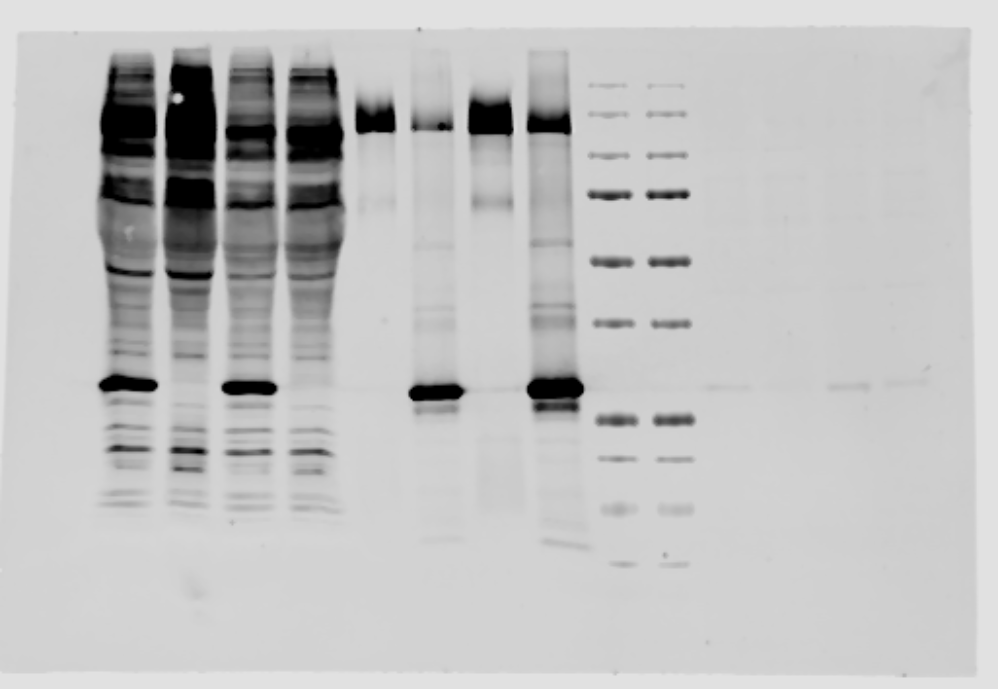

Supplement: Source data 2. [file elife-73611-data2.zip › raw original data/Figure 2 Source data raw panel A 1.tif]

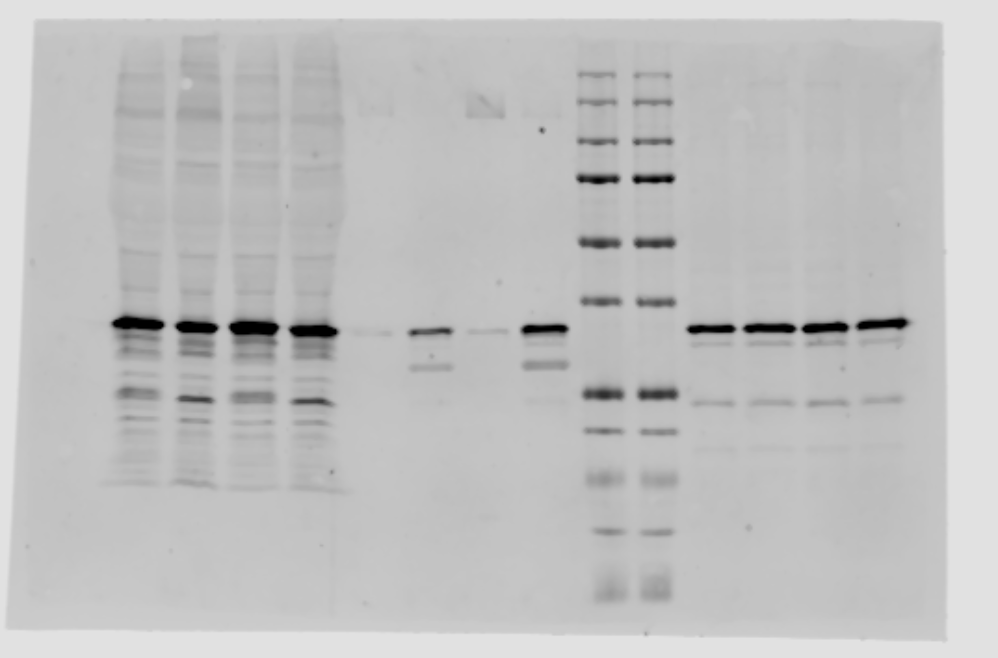

Supplement: Source data 2. [file elife-73611-data2.zip › raw original data/Figure 2 Source data raw panel A 2.png]

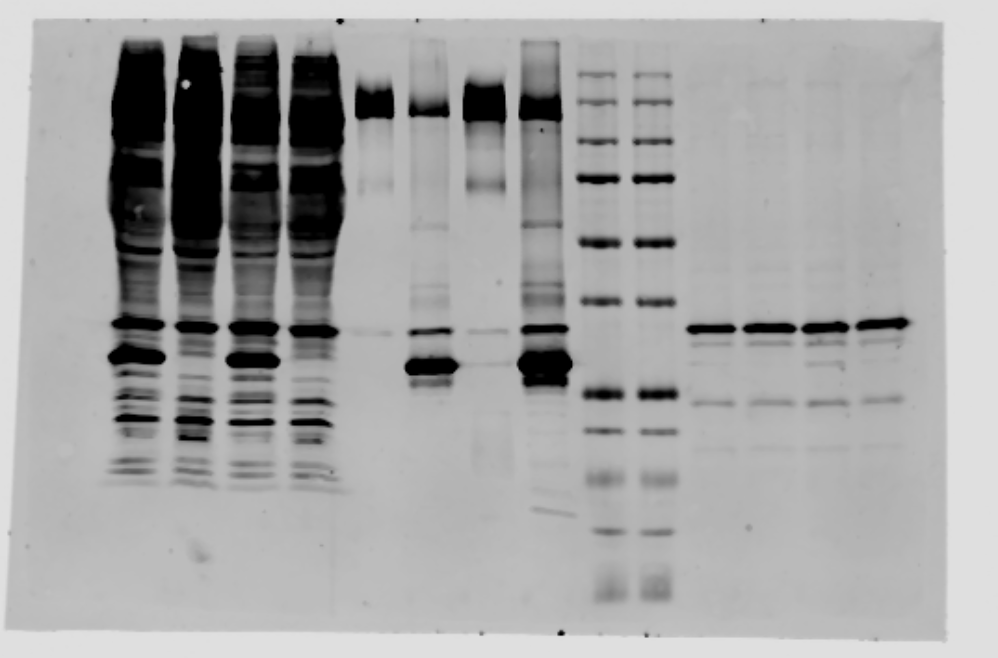

Supplement: Source data 2. [file elife-73611-data2.zip › raw original data/Figure 2 Source data raw panel A 3.tif]

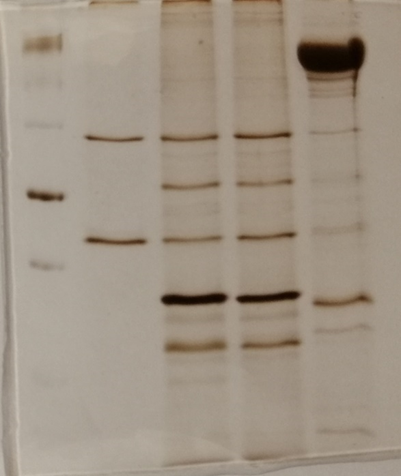

Supplement: Source data 2. [file elife-73611-data2.zip › raw original data/Figure 2 Source data raw panel B 1.tif]

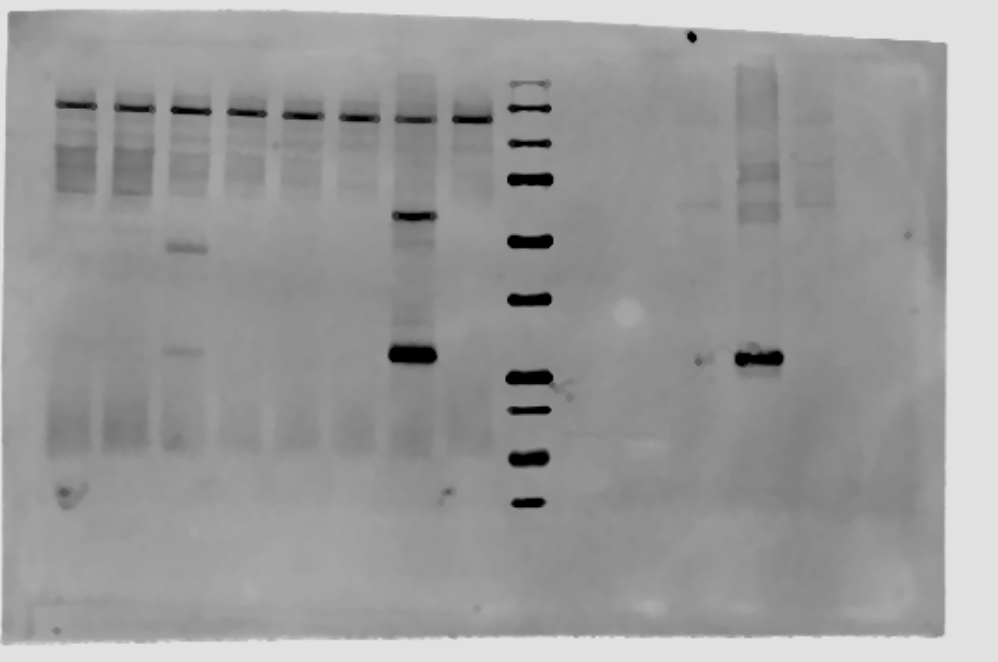

Supplement: Source data 2. [file elife-73611-data2.zip › raw original data/Figure 2 Source data raw panel B 2.tif]

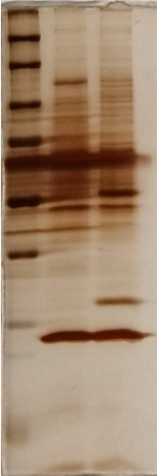

Supplement: Source data 2. [file elife-73611-data2.zip › raw original data/Figure 2 Source data raw panel C 2.tif]

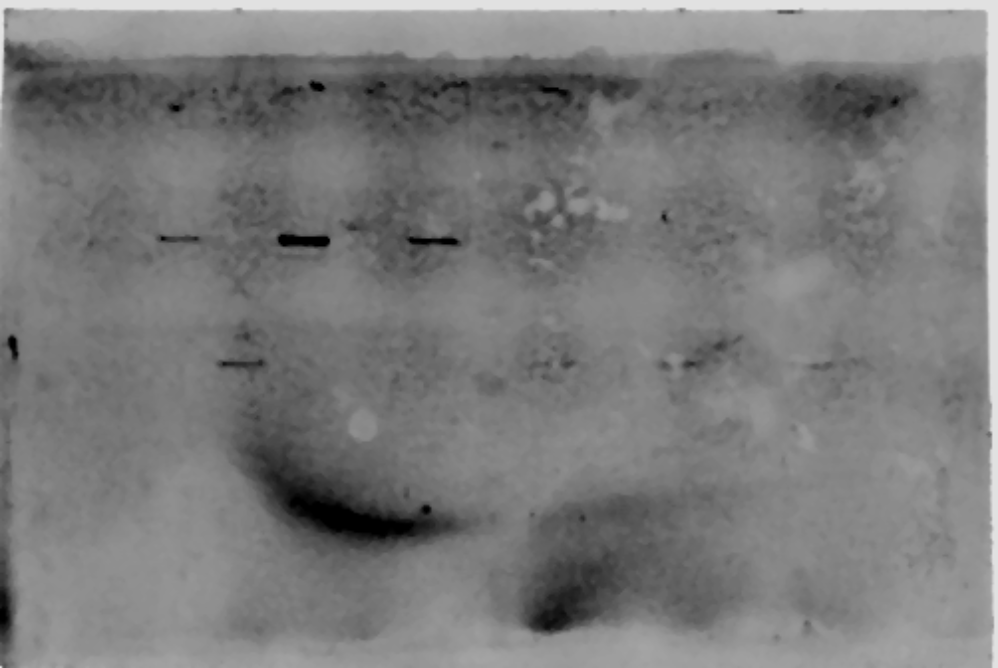

Supplement: Source data 2. [file elife-73611-data2.zip › raw original data/Figure 2 Source data raw panel D 1.tif]

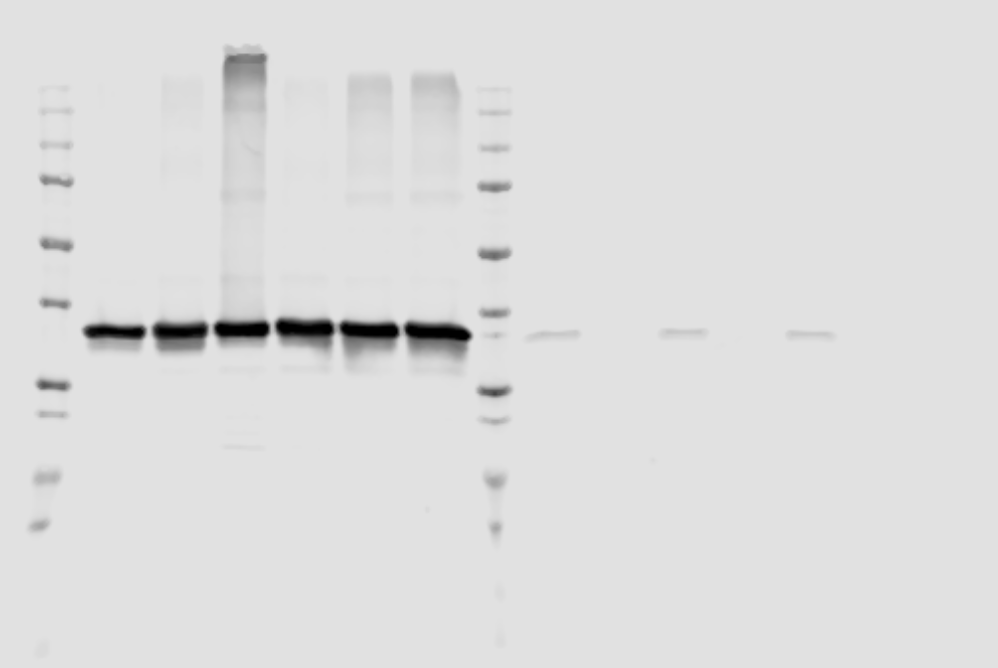

Supplement: Source data 2. [file elife-73611-data2.zip › raw original data/Figure 2 Source data raw panel D 2.tif]

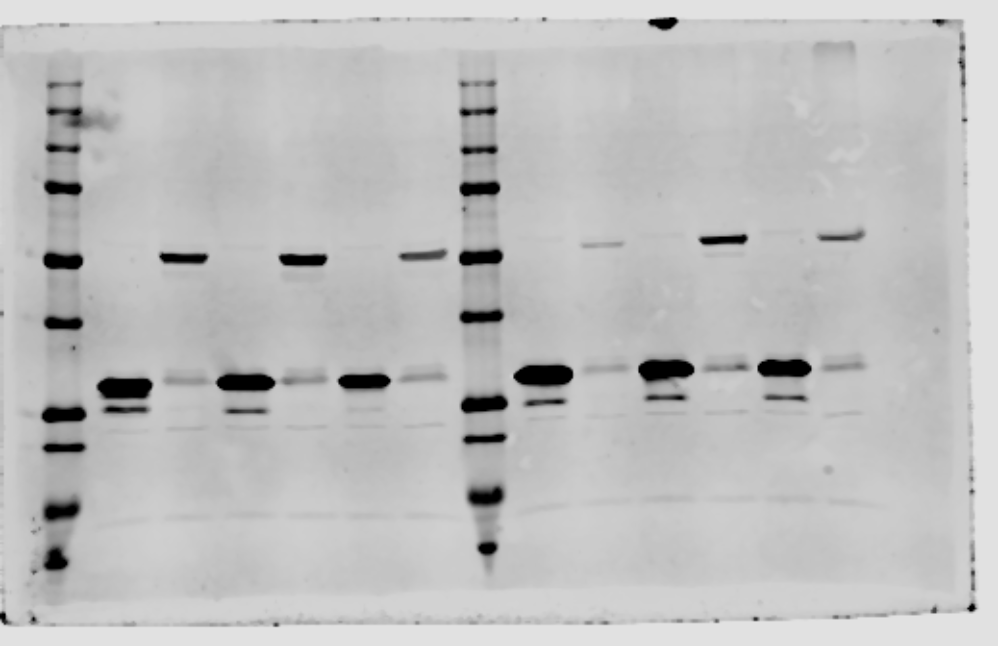

Supplement: Source data 2. [file elife-73611-data2.zip › raw original data/Figure 2 Source data raw panel D 3.tif]

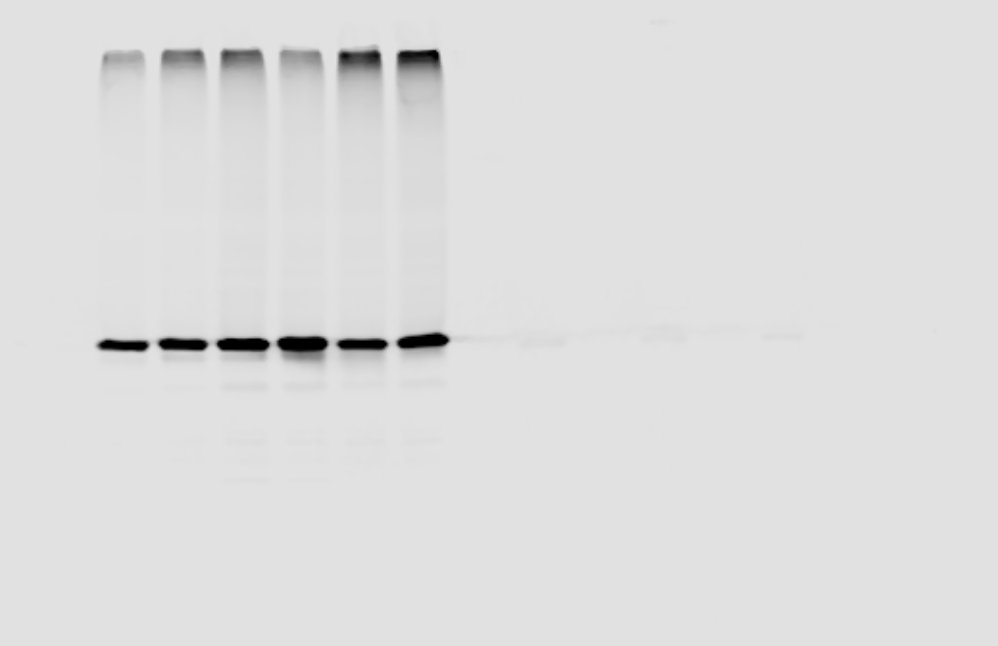

Supplement: Source data 2. [file elife-73611-data2.zip › raw original data/Figure 2 Source data raw panel D 4.tif]

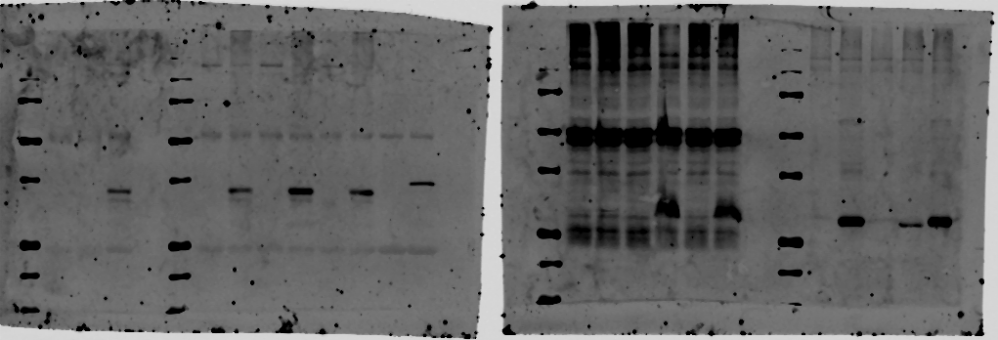

Supplement: Source data 2. [file elife-73611-data2.zip › raw original data/Figure 3 Source data raw panel A 1.tif]

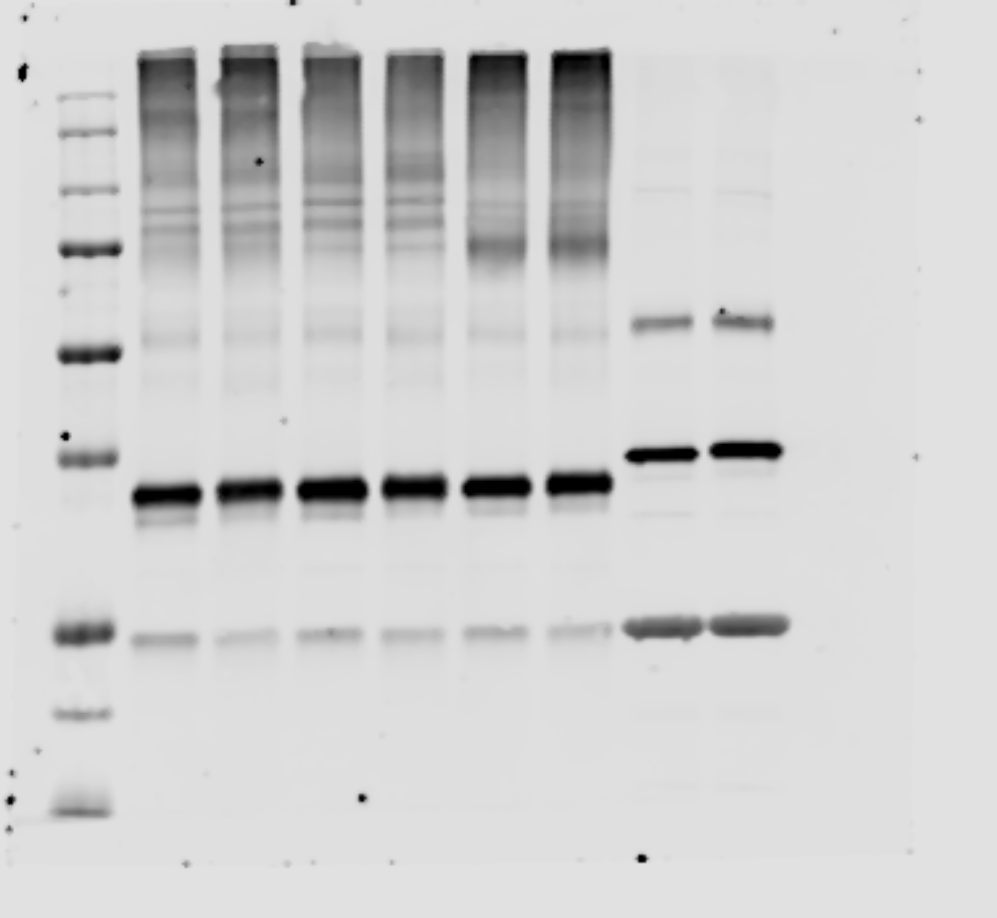

Supplement: Source data 2. [file elife-73611-data2.zip › raw original data/Figure 3 Source data raw panel A 2.tif]

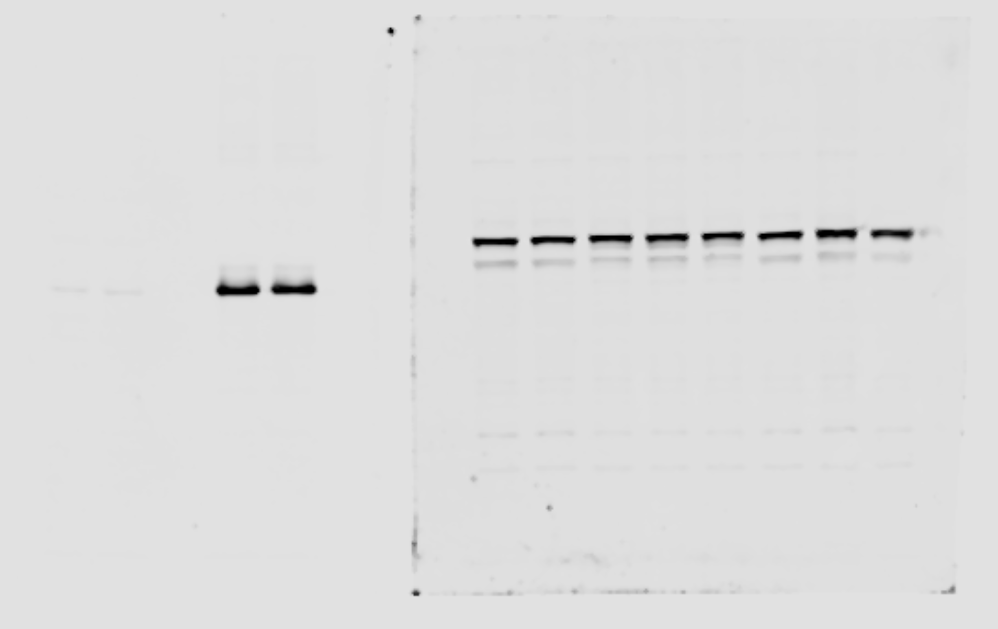

Supplement: Source data 2. [file elife-73611-data2.zip › raw original data/Figure 3 Source data raw panel A 3.tif]

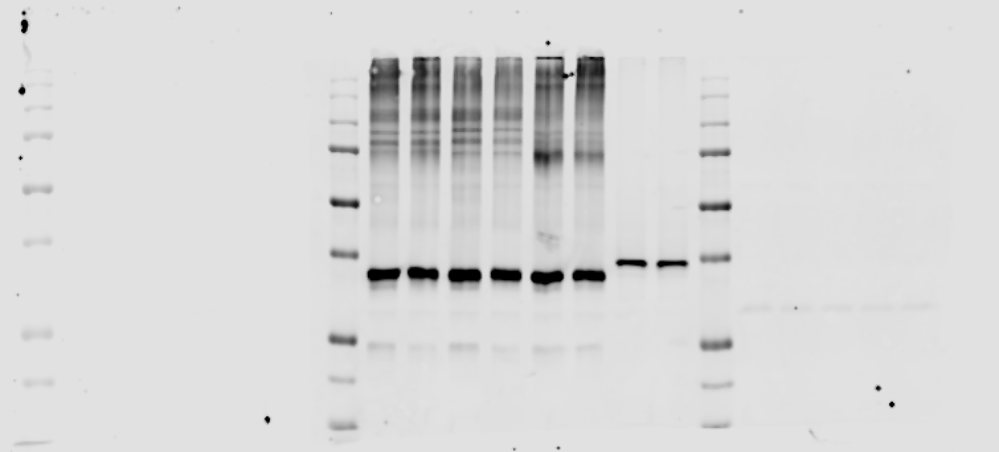

Supplement: Source data 2. [file elife-73611-data2.zip › raw original data/Figure 3 Source data raw panel A 4.tif]

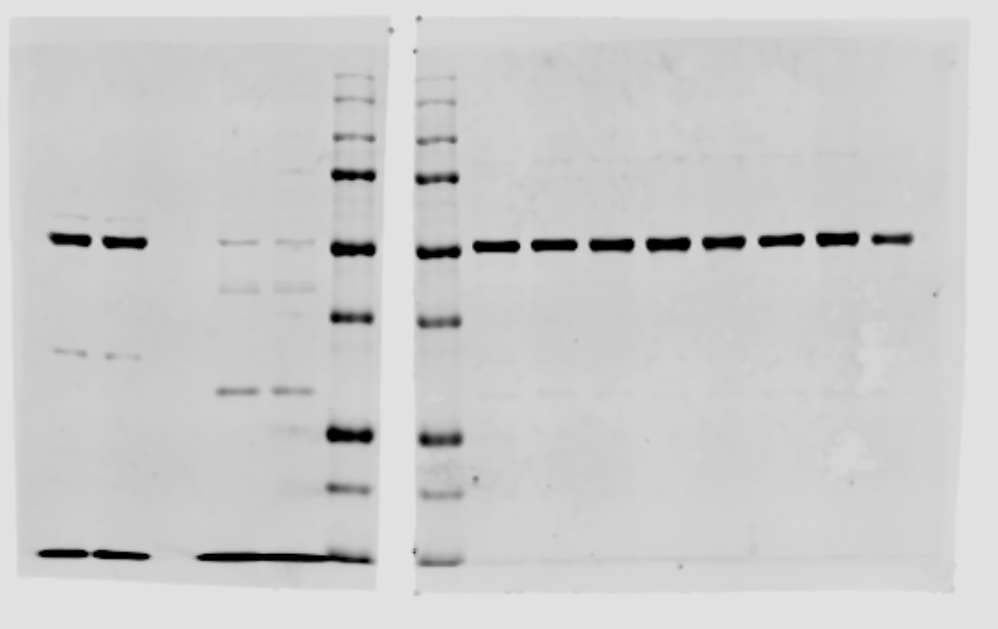

Supplement: Source data 2. [file elife-73611-data2.zip › raw original data/Figure 3 Source data raw panel A 5.tif]

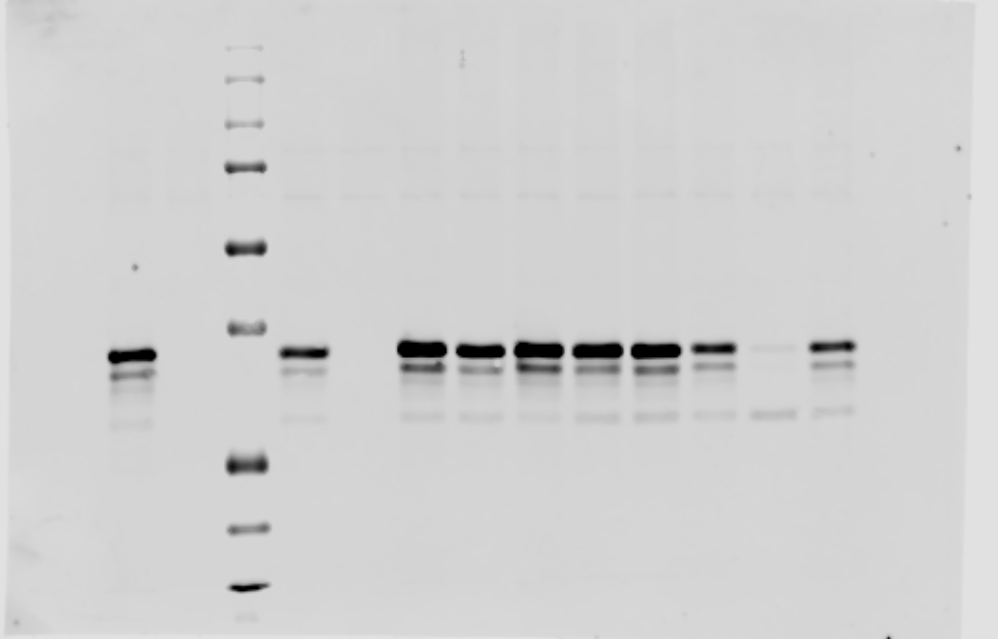

Supplement: Source data 2. [file elife-73611-data2.zip › raw original data/Figure 3 Source data raw panel D 1.tif]

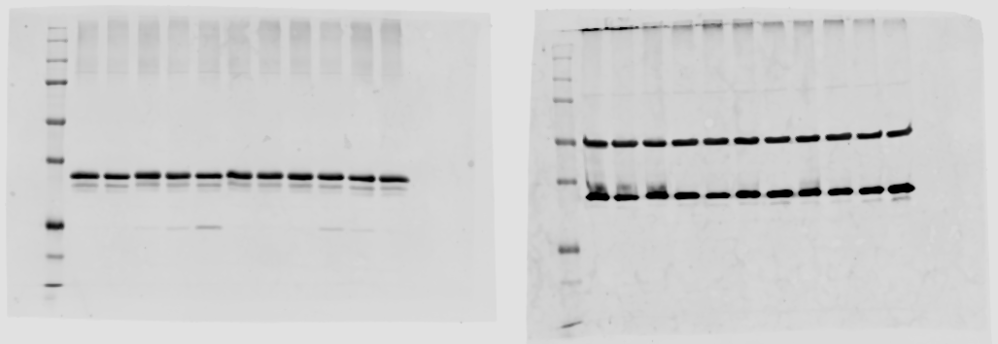

Supplement: Source data 2. [file elife-73611-data2.zip › raw original data/Figure 3 Source data raw panel D 2.tif]

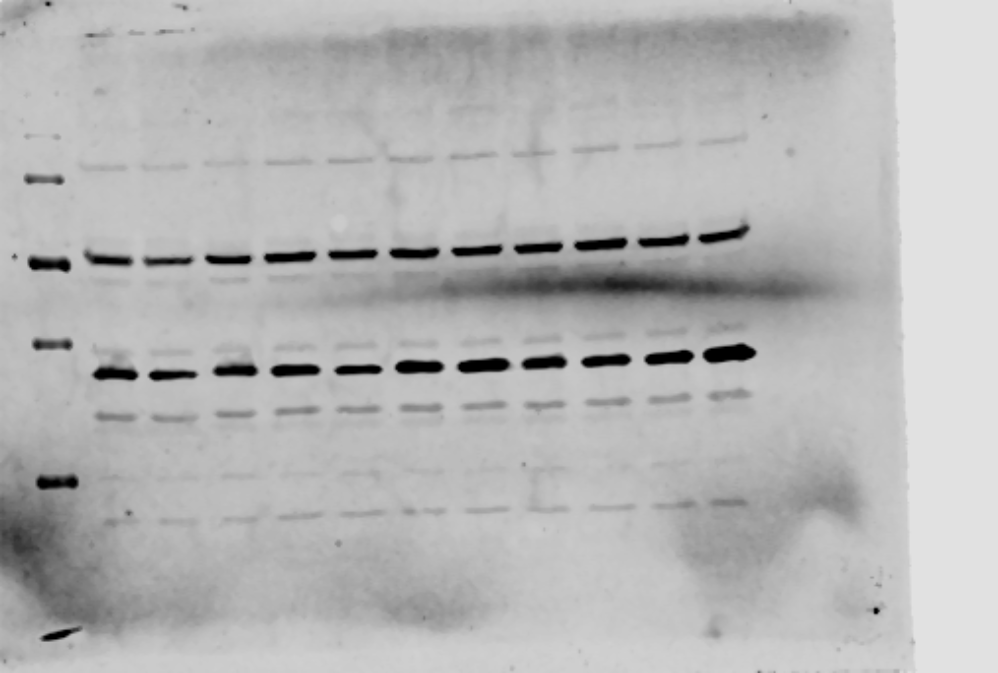

Supplement: Source data 2. [file elife-73611-data2.zip › raw original data/Figure 3 Source data raw panel D 3.tif]

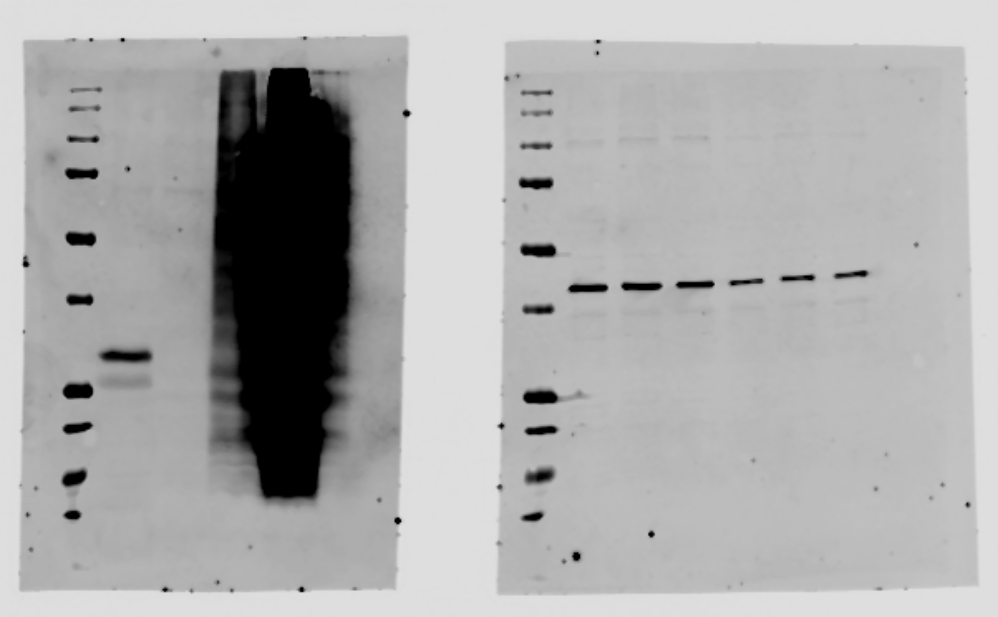

Supplement: Source data 2. [file elife-73611-data2.zip › raw original data/Figure 3 Source data raw panel E 1.tif]

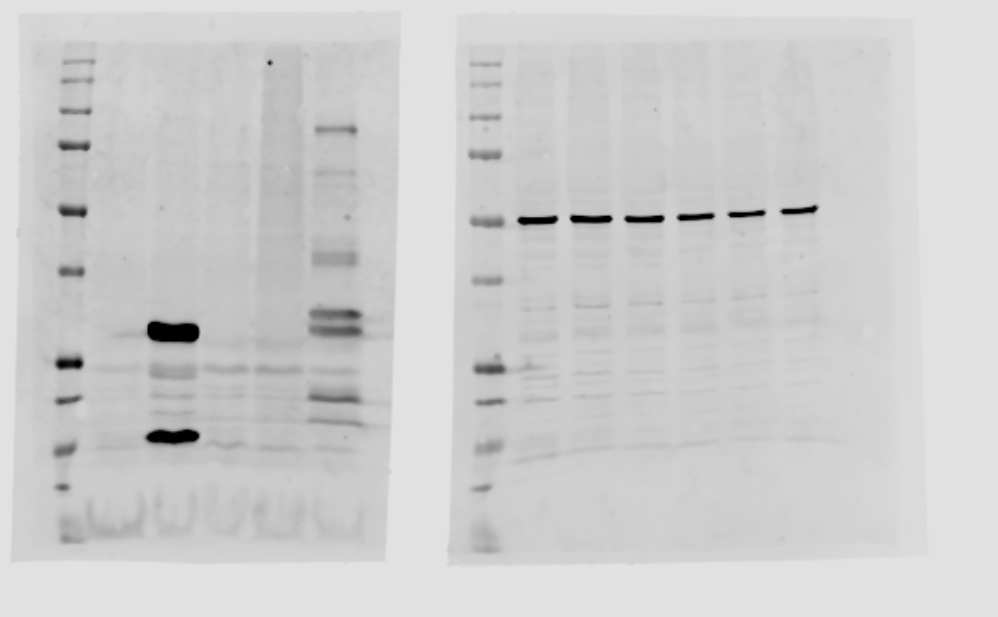

Supplement: Source data 2. [file elife-73611-data2.zip › raw original data/Figure 3 Source data raw panel E 2.tif]

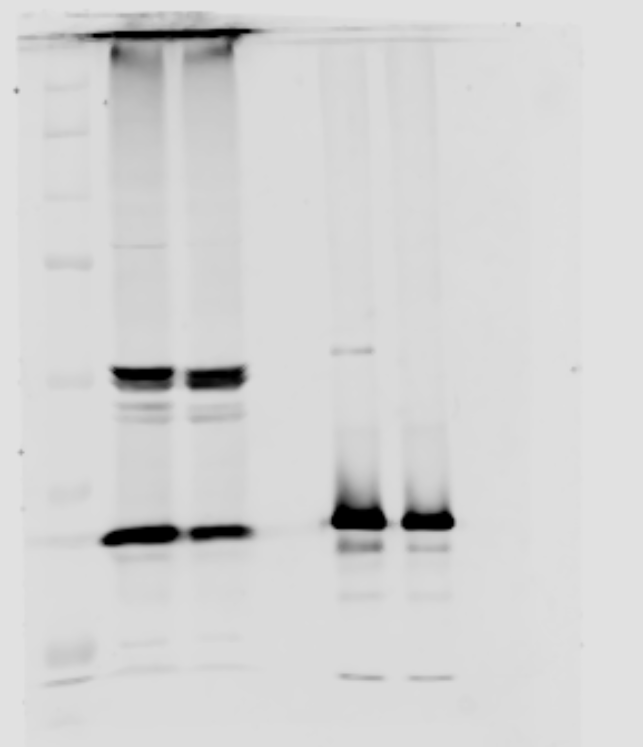

Supplement: Source data 2. [file elife-73611-data2.zip › raw original data/Figure 3-figure supplement 1 Source data panel A 2.tif]

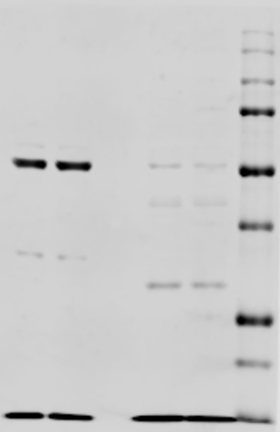

Supplement: Source data 2. [file elife-73611-data2.zip › raw original data/Figure 3-figure supplement 1 Source data panel A 3.tif]

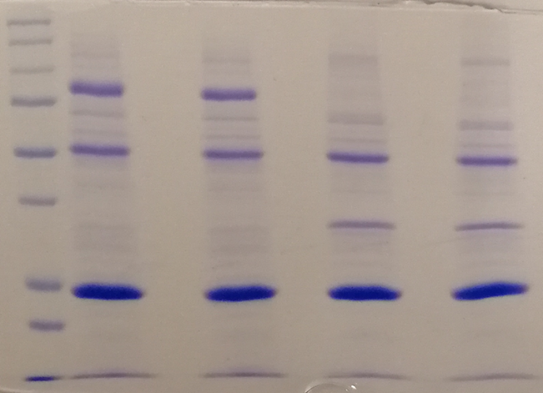

Supplement: Source data 2. [file elife-73611-data2.zip › raw original data/Figure 3-figure supplement 1 Source data panel B.png]

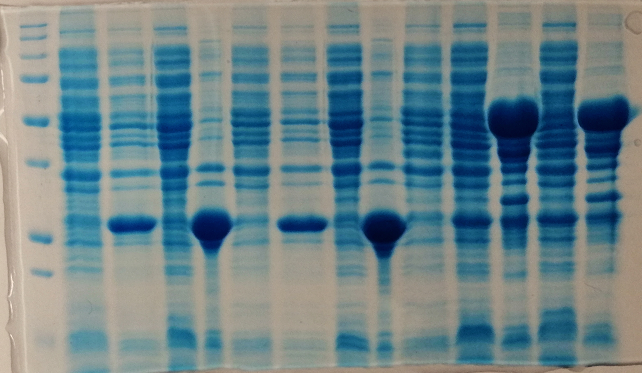

Supplement: Source data 2. [file elife-73611-data2.zip › raw original data/Figure 3-figure supplement 3 Source data panel A 1.png]

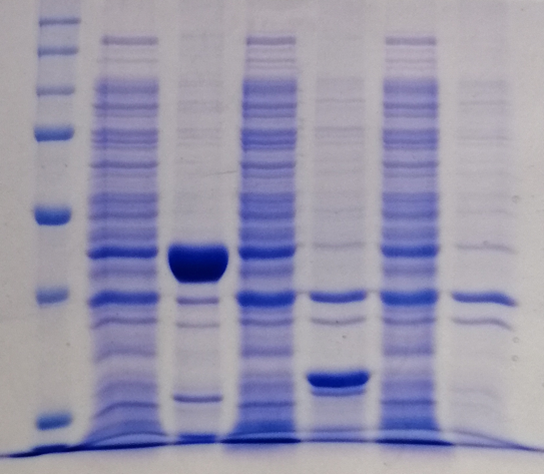

Supplement: Source data 2. [file elife-73611-data2.zip › raw original data/Figure 3-figure supplement 3 Source data panel A 2.png]

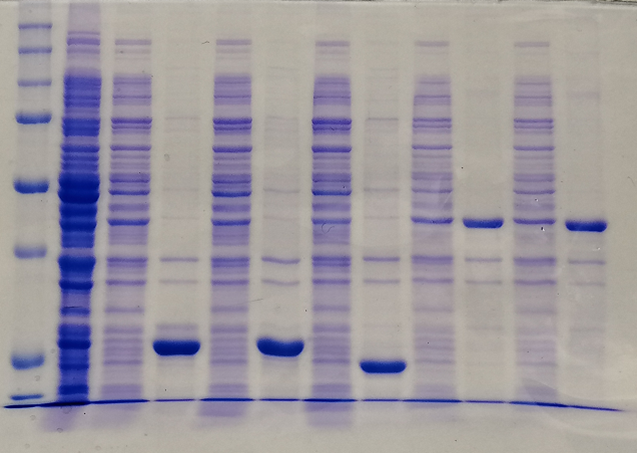

Supplement: Source data 2. [file elife-73611-data2.zip › raw original data/Figure 3-figure supplement 3 Source data panel B 1.png]

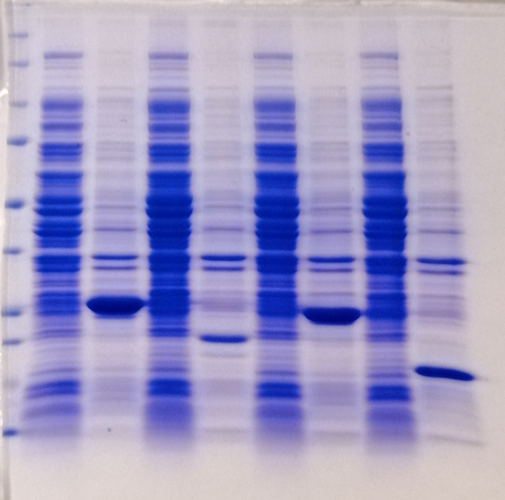

Supplement: Source data 2. [file elife-73611-data2.zip › raw original data/Figure 3-figure supplement 3 Source data panel B 2.png]

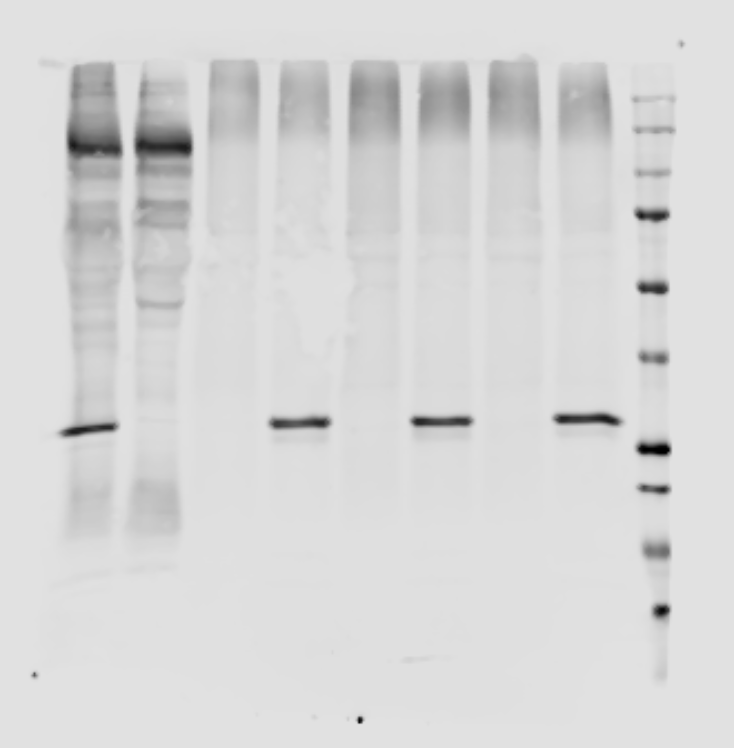

Supplement: Source data 2. [file elife-73611-data2.zip › raw original data/Figure 3-figure supplement 4 Source data panel A 1.tif]

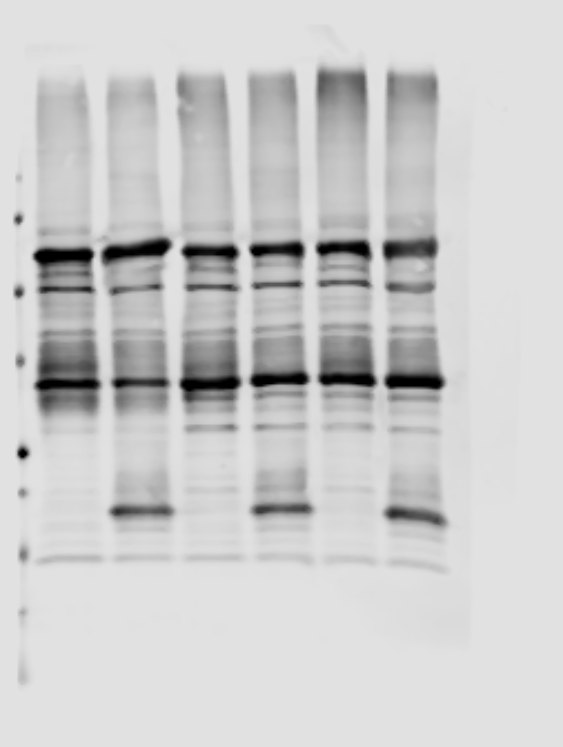

Supplement: Source data 2. [file elife-73611-data2.zip › raw original data/Figure 3-figure supplement 4 Source data panel A 2.tif]

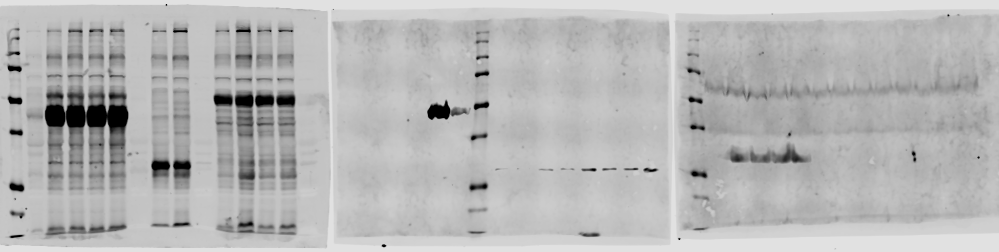

Supplement: Source data 2. [file elife-73611-data2.zip › raw original data/Figure 3-figure supplement 4 Source data panel B 1.tif]

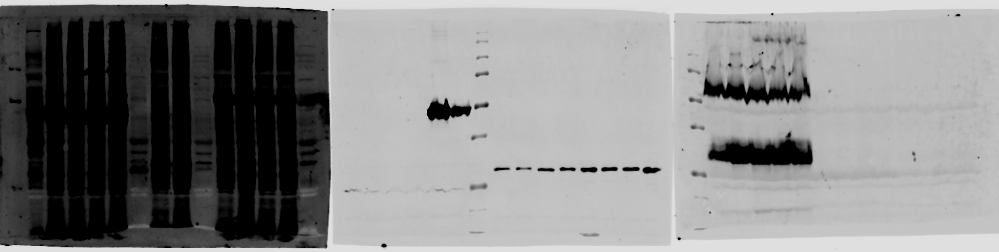

Supplement: Source data 2. [file elife-73611-data2.zip › raw original data/Figure 3-figure supplement 4 Source data panel B 2.tif]

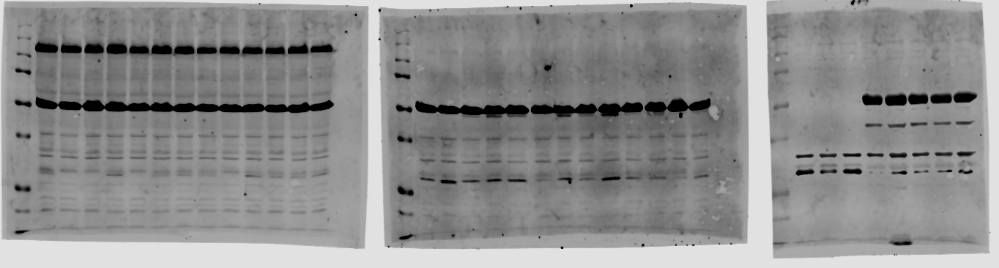

Supplement: Source data 2. [file elife-73611-data2.zip › raw original data/Figure 3-figure supplement 4 Source data panel B 3.tif]

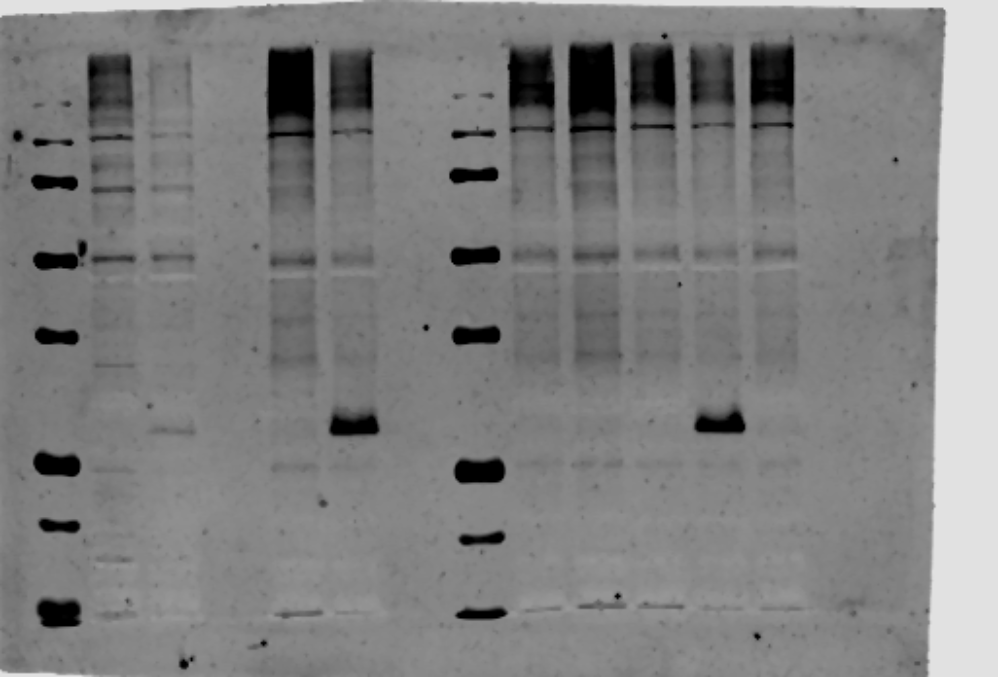

Supplement: Source data 2. [file elife-73611-data2.zip › raw original data/Figure 4 Source data raw panel A 1.tif]

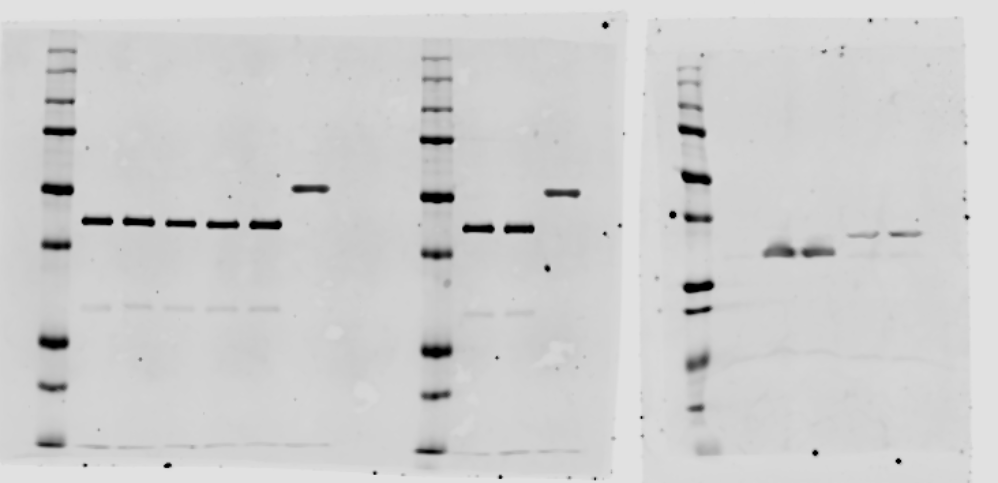

Supplement: Source data 2. [file elife-73611-data2.zip › raw original data/Figure 4 Source data raw panel A 2.tif]

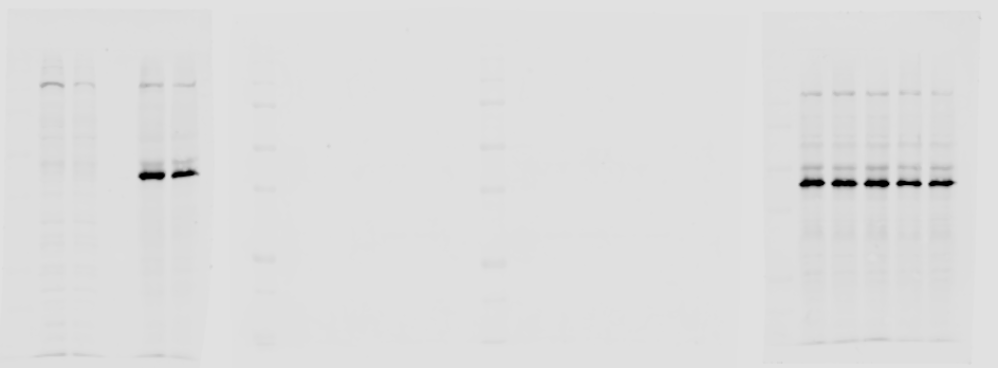

Supplement: Source data 2. [file elife-73611-data2.zip › raw original data/Figure 4 Source data raw panel A 3.tif]

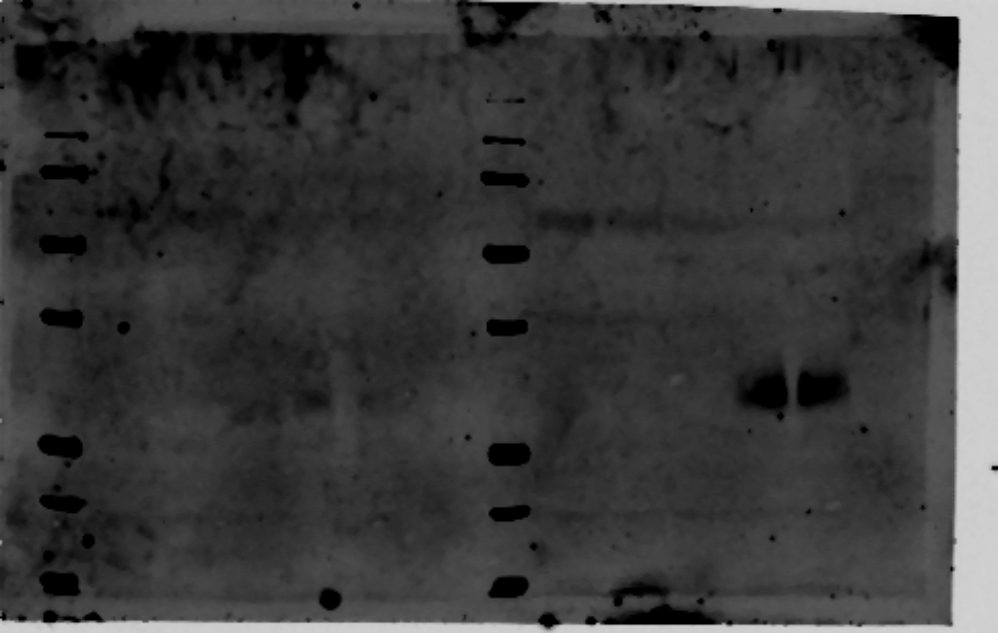

Supplement: Source data 2. [file elife-73611-data2.zip › raw original data/Figure 4 Source data raw panel A 4.tif]

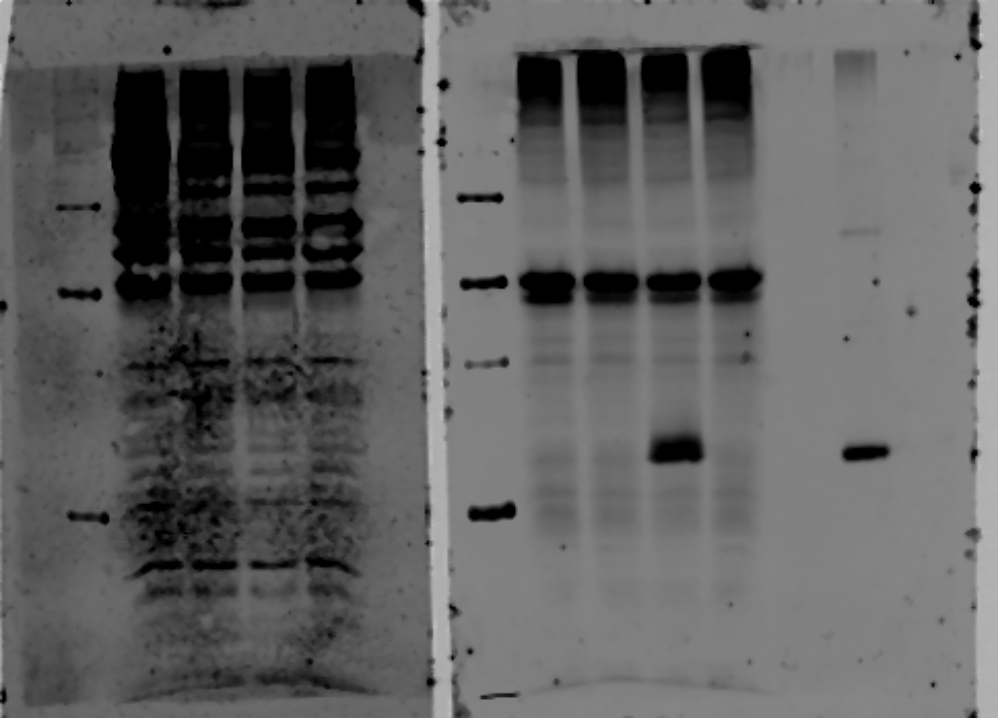

Supplement: Source data 2. [file elife-73611-data2.zip › raw original data/Figure 4 Source data raw panel B 1.tif]

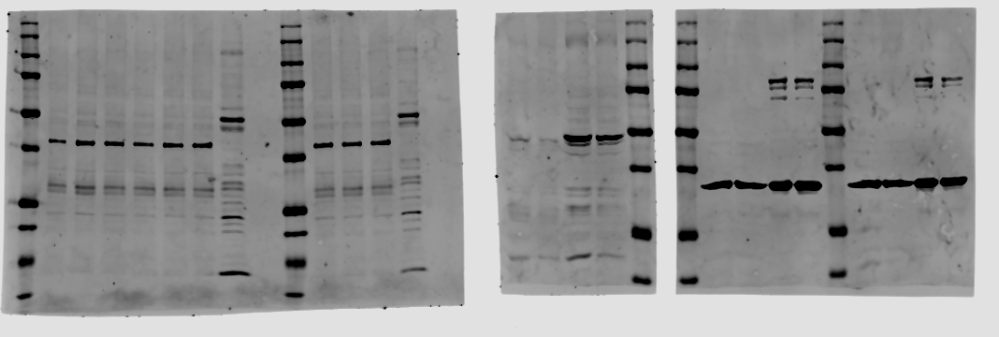

Supplement: Source data 2. [file elife-73611-data2.zip › raw original data/Figure 4 Source data raw panel B 2.tif]

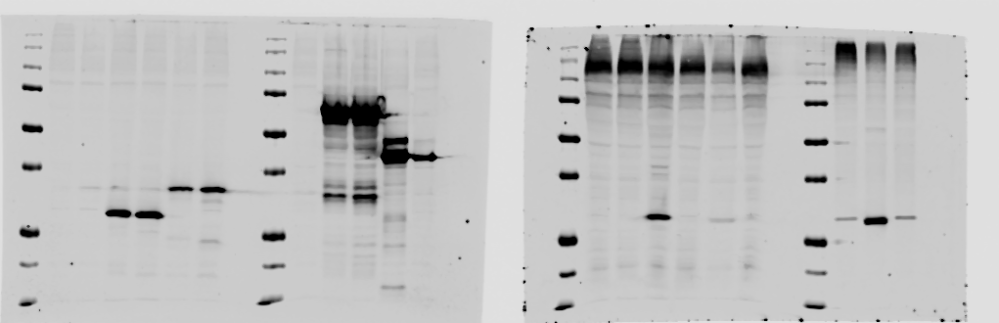

Supplement: Source data 2. [file elife-73611-data2.zip › raw original data/Figure 4 Source data raw panel B 3.tif]

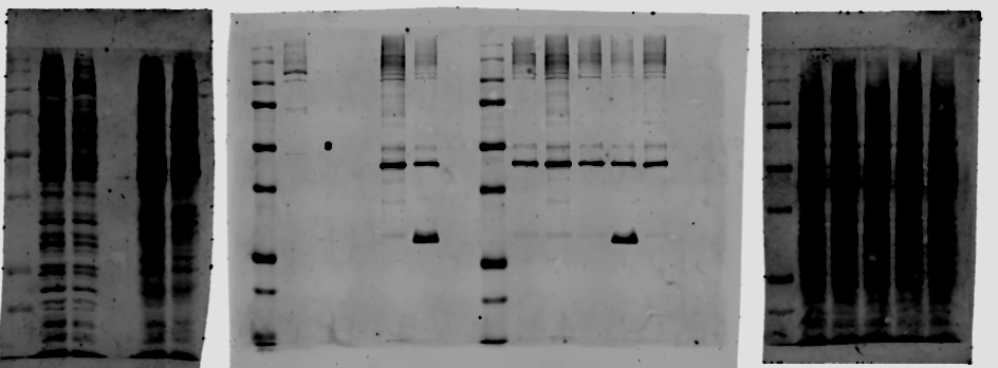

Supplement: Source data 2. [file elife-73611-data2.zip › raw original data/Figure 4 Source data raw panel B 4.tif]

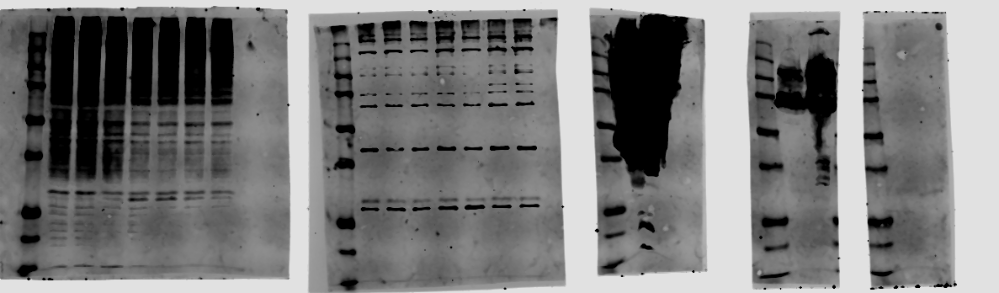

Supplement: Source data 2. [file elife-73611-data2.zip › raw original data/Figure 4 Source data raw panel C 1.tif]

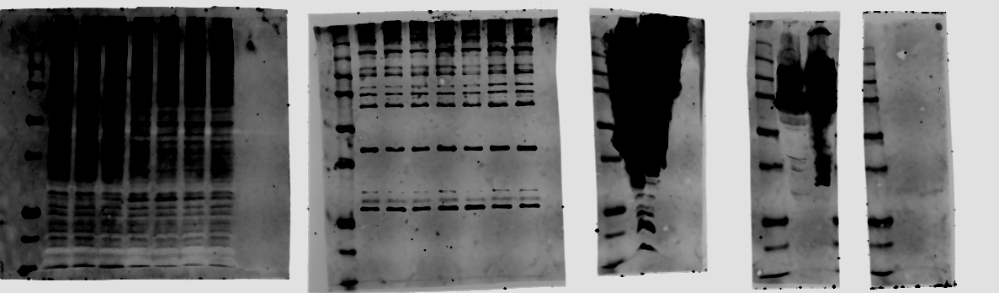

Supplement: Source data 2. [file elife-73611-data2.zip › raw original data/Figure 4 Source data raw panel C 2.tif]

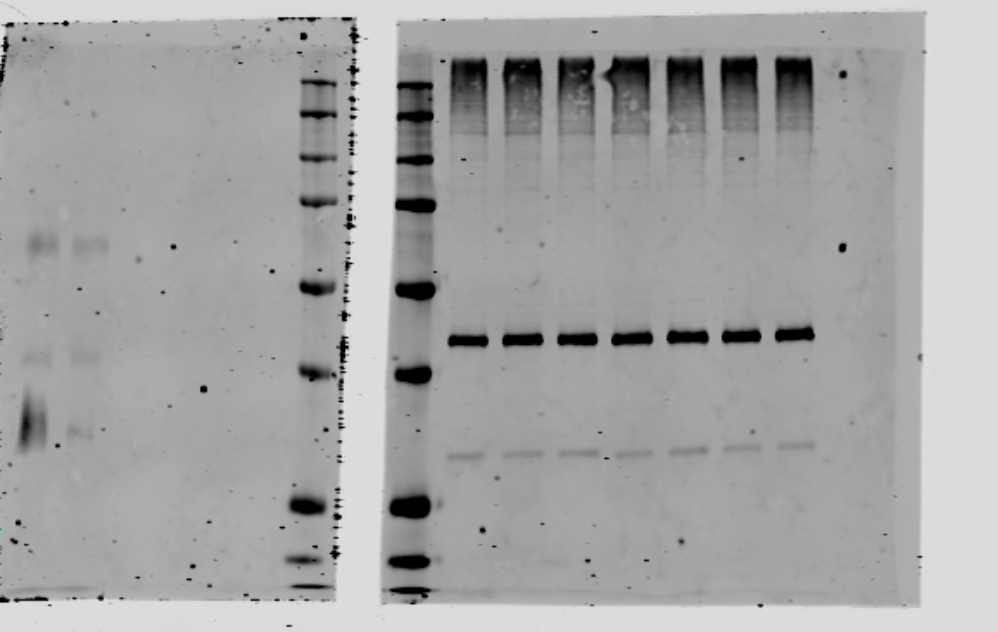

Supplement: Source data 2. [file elife-73611-data2.zip › raw original data/Figure 4 Source data raw panel C 3.tif]

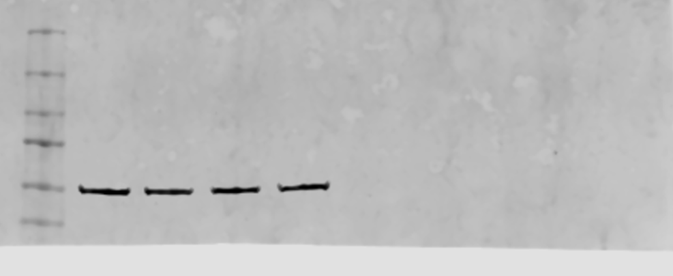

Supplement: Source data 2. [file elife-73611-data2.zip › raw original data/Figure 5-figure supplement 1 Source data panel B 1.png]

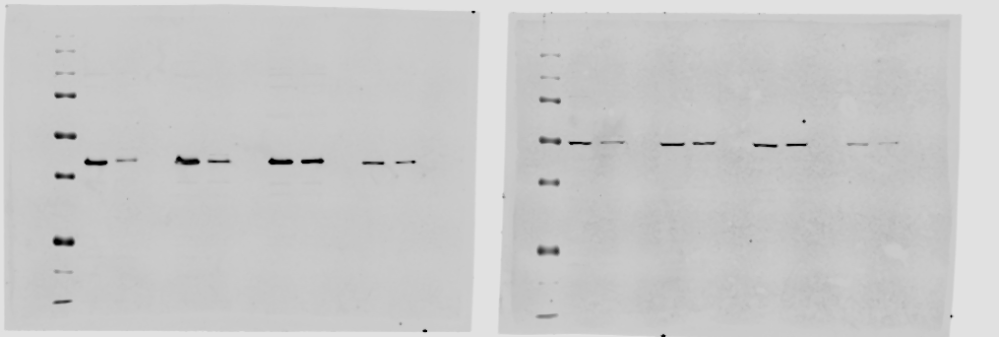

Supplement: Source data 2. [file elife-73611-data2.zip › raw original data/Figure 5-figure supplement 1 Source data panel A 1.tif]

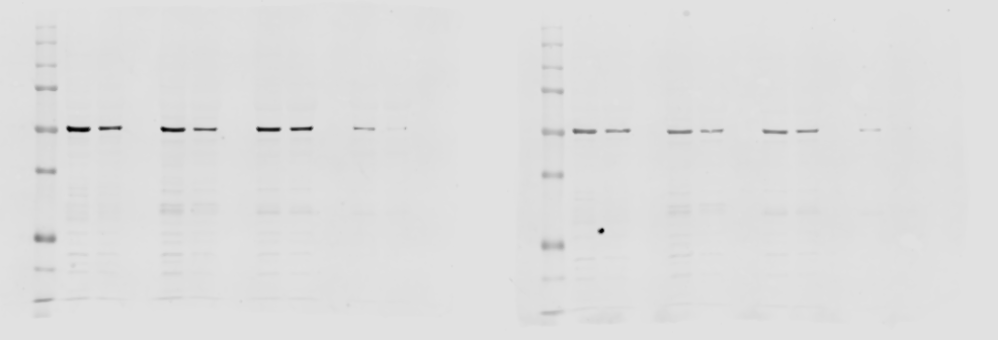

Supplement: Source data 2. [file elife-73611-data2.zip › raw original data/Figure 5-figure supplement 1 Source data panel A 2.tif]

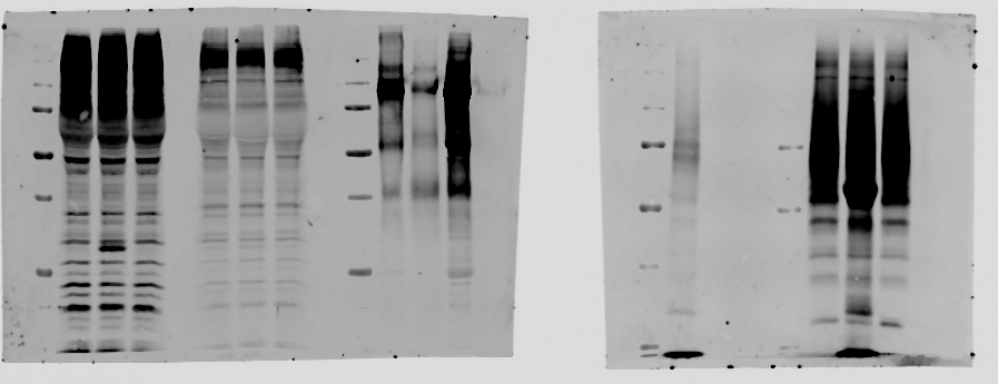

Supplement: Source data 2. [file elife-73611-data2.zip › raw original data/Figure 5-figure supplement 1 Source data panel B 1.tif]

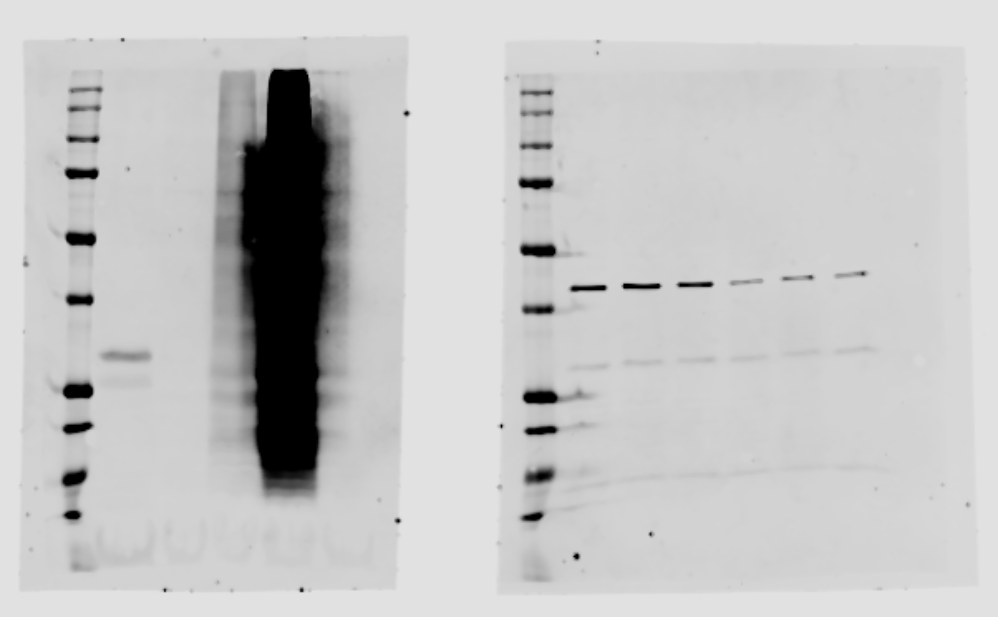

Supplement: Source data 2. [file elife-73611-data2.zip › raw original data/Figure 5-figure supplement 1 Source data panel C 1.tif]
